# Consequences of blunting the mevalonate pathway in cancer identified by a pluri-omics approach

**DOI:** 10.1038/s41419-018-0761-0

**Published:** 2018-07-03

**Authors:** Sophie Goulitquer, Mikaël Croyal, Julie Lalande, Anne-Lise Royer, Yann Guitton, Danielle Arzur, Stéphanie Durand, Catherine Le Jossic-Corcos, Alain Bouchereau, Philippe Potin, Serge Akoka, Jean-Philippe Antignac, Michel Krempf, Véronique Ferchaud-Roucher, Patrick Giraudeau, Laurent Corcos

**Affiliations:** 1 Génétique, Génomique Fonctionnelle et Biotechnologies, INSERM, Université de Brest, EFS, Brest, France; 2 INRA UMR 1280, Centre de Recherche en Nutrition Humaine de l’Ouest (CRNHO), CHU Hôtel-Dieu, Université de Nantes, Nantes, France; 3 CEISAM UMR 6230, EBSI Team, Université de Nantes, CNRS, Nantes, France; 4 Laboratoire d’Etude des Résidus et Contaminants dans les aliments (LABERCA), Ecole Nationale Vétérinaire, Agroalimentaire et de l’Alimentation Nantes Atlantique (Oniris), Nantes, France; 5 Institut de Génétique, Environnement et Protection des Plantes, UMR 1349 INRA-Agrocampus Ouest-Université de Rennes 1, Rennes-Le Rheu, France; 6 Team Biochemistry of Algal Defenses, UMR 7139 CNRS-UPMC & LIA-DIAMS, Station Biologique, Roscoff, France; 7 Institut Universitaire de France, Paris, France

## Abstract

We have previously shown that the combination of statins and taxanes was a powerful trigger of HGT-1 human gastric cancer cells’ apoptosis^1^. Importantly, several genes involved in the “Central carbon metabolism pathway in cancer”, as reported in the Kyoto Encyclopedia of Genes and Genomes, were either up- (*ACLY*, *ERBB2, GCK, MYC, PGM, PKFB2, SLC1A5, SLC7A5, SLC16A3,)* or down- *(IDH, MDH1, OGDH, P53, PDK*) regulated in response to the drug association. In the present study, we conducted non-targeted metabolomics and lipidomics analyses by complementary methods and cross-platform initiatives, namely mass spectrometry (GC-MS, LC-MS) and nuclear magnetic resonance (NMR), to analyze the changes resulting from these treatments. We identified several altered biochemical pathways involved in the anabolism and disposition of amino acids, sugars, and lipids. Using the Cytoscape environment with, as an input, the identified biochemical marker changes, we distinguished the functional links between pathways. Finally, looking at the overlap between metabolomics/lipidomics and transcriptome changes, we identified correlations between gene expression modifications and changes in metabolites/lipids. Among the metabolites commonly detected by all types of platforms, glutamine was the most induced (6–7-fold), pointing to an important metabolic adaptation of cancer cells. Taken together, our results demonstrated that combining robust biochemical and molecular approaches was efficient to identify both altered metabolic pathways and overlapping gene expression alterations in human gastric cancer cells engaging into apoptosis following blunting the cholesterol synthesis pathway.

## Introduction

Cancer cell metabolism shows strong alterations required for cell activity and growth^2^, including a high avidity for glucose and lipids^3–5^. Statins, cholesterol-lowering drugs used to prevent cardiovascular diseases, are competitive inhibitors of 3-hydroxy-3-methyl-glutaryl-coenzyme A reductase (HMG-CoA Red). They block mevalonate production, in a rate-limiting step of the cholesterol synthesis pathway^6^. In addition, statins induce apoptosis of many cancer cell types, an effect counteracting the addiction of cancer cells to pathways driving cell division, motility, and proliferation^7^. Indeed, inhibition of HMG-CoA Red results in a shortage of several important metabolites, including Farnesyl PyroPhosphate (FPP) and GeranylGeranyl PyroPhosphate (GGPP)^7,8^. FPP and GGPP bind, in a post-translational manner (prenylation), to the C-terminus of a restricted set of proteins from the Ras and Rho families, which makes those proteins migrate and anchor to the plasma membrane, where they acquire GTPase activity. Prenylation blockade results in restriction of the positive growth signals associated with the MAP kinase-dependent pathways^9^. Docetaxel, an anticancer taxane compound used to treat breast and gastric cancer^10^, promotes microtubules assembly and stabilizes the polymers against depolymerization, thereby inhibiting microtubule dynamics^11^.

We reported that both lovastatin and docetaxel triggered efficient apoptosis of HGT-1 human gastric cancer cells, but also colon or liver cancer cells^1^. The combination of lovastatin and docetaxel resulted in a synergistic apoptotic effect, as compared to either compound alone. Furthermore, supplementing adenocarcinoma cells with FFP or GGPP prevented statin-dependent apoptosis of several cancer cell types, including gastric cancer cells^12^. Finally, we showed that lovastatin triggered numerous gene expression changes, whereas docetaxel had little effects^1^.

Based on these results, we decided to investigate the potential of these drugs to alter the metabolomics and lipidomics of early cancer cell engagement into apoptosis. Such markers would hold promise as a characteristic signature of changes resulting from treatment with these drugs and, potentially, with other cytotoxic drugs. To this end, we embarked on a collaborative project with mass spectrometry (GC-MS and LC-MS) and Nuclear Magnetic Resonance (NMR) platforms from the CORSAIRE metabolomics and lipidomics network (https://www.biogenouest.org/en), in a non-targeted approach, to determine the modifications of metabolomics and lipidomics profiles in HGT-1 cells. The levels of more than 100 metabolites and lipids were significantly altered by the treatments, including amino acids, organic acids, sugars, and several families of lipids. Furthermore, we identified a small set of compounds with altered accumulation levels jointly recognized by all analytical platforms, thereby conferring robustness to our methodological framework. Strikingly, most changes were due to lovastatin treatment, used alone or with docetaxel, which had little effect by itself. Finally, we combined metabolomics/lipidomics and transcriptomics data obtained from lovastatin and/or docetaxel-treated cells, which pointed to key biochemical alterations and to the recognition of novel biomarkers of the onset of cancer cell apoptosis.

## Results

All experiments were performed with extracts from cells treated by the drugs for 36 h, with the exception of the kinetics experiments (17, 24, and 36 h).

### HMG-CoA reductase enzyme activity following drug treatments

In order to evaluate the effects of lovastatin and/or docetaxel on HMG-CoA Red activity, we determined the level of mevalonate produced. As expected, any condition that included lovastatin resulted in full absence of HMG-CoA Red activity. The basal enzyme activity was 18 ± 2 pmol/min/mg protein. Docetaxel had no effect on this basal activity, and did not overcome the suppression resulting from lovastatin.

### Cross-platform principal component analysis

**Fig. 1:**
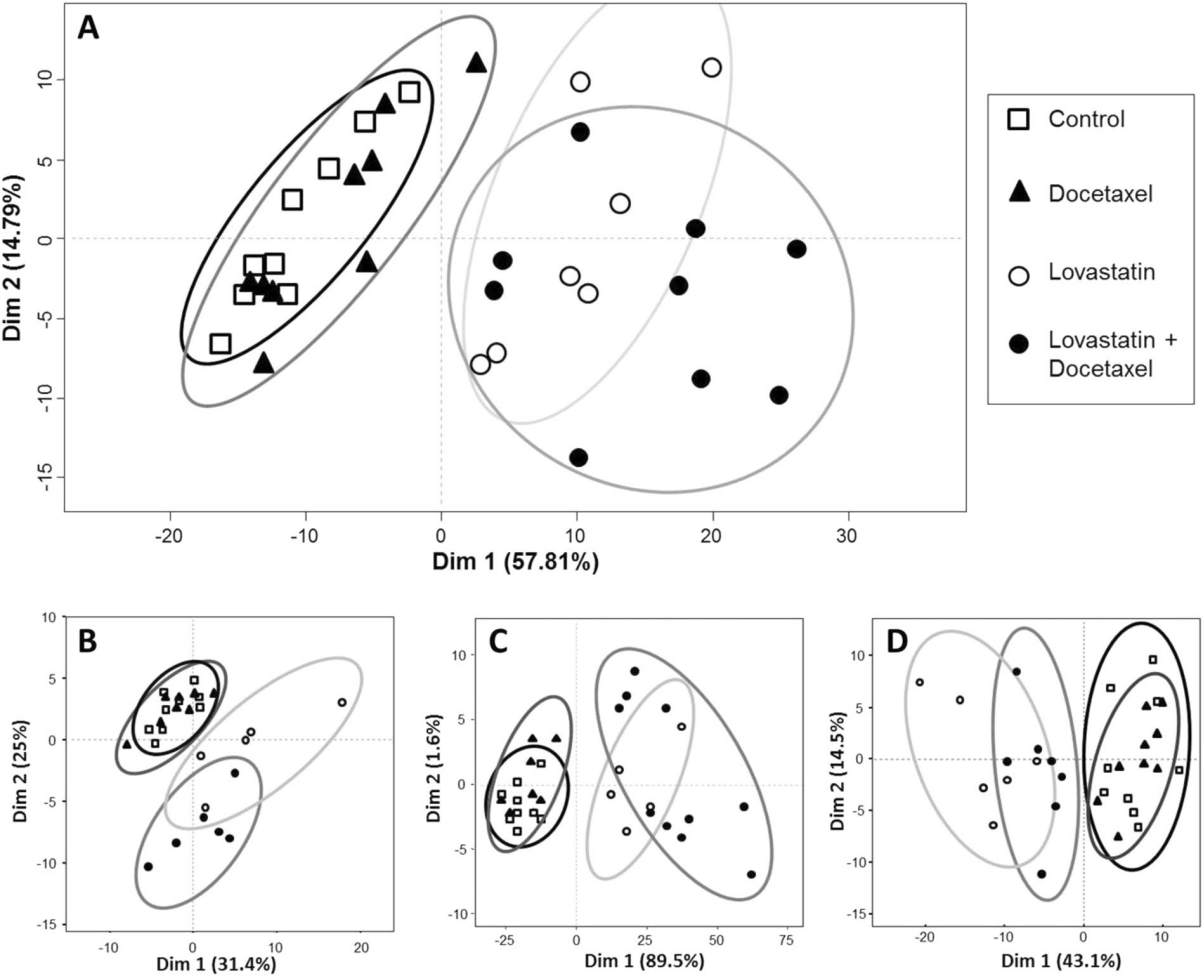
**Representative score plots of the Principal Component Analysis (PCA) from metabolomics and lipidomics analyses**. Score plots of the metabolomics MS data acquired in the negative mode on the LC-MSn°2 platform (A), the LC-MSn°1 platform in the positive mode (B), the GC-MSn°1 platform (C), and the NMRn°1 platform (D). Experimental conditions: control (open squares), docetaxel (plain triangles), lovastatin (open circles), and lovastatin + docetaxel (plain circles). Cells were treated for 36 h. The two dimensions of the analysis and their contribution to the variance are indicated. The data are representative of results from all platforms

In order to see if the treatment of HGT-1 cells by lovastatin and/or docetaxel modified the metabolome and lipidome, we performed Principal Component Analyses (PCA) using, as a first entry, harmonized metabolomics data following Pareto and log10 transformation to have data from each platform within a similar order of magnitude^13^. No marker selection was performed upstream of the PCA analysis. In addition, a PCA analysis was conducted before and after normalization, which showed that the normalization step did not modify the overall discriminatory effects of the treatments. A strong separation was observed between conditions with lovastatin and without lovastatin in HGT-1 (Fig. 1) but also in AGS human gastric cancer and HCT116 human colon carcinoma cells (Supplementary Figure 1).

Because of their distinct modes of action, lovastatin, and docetaxel were not necessarily expected to both trigger large and convergent metabolomics/lipidomics changes. Whereas docetaxel acts mechanically onto microtubule dynamics and induced only a few gene expression changes, lovastatin strongly altered several gene expression programs^1^. Although the metabolome/lipidome changes may not necessarily converge onto common gene expression-driven alterations, this observation could explain our results, at least in part.

### Comparison of metabolomics data among analytical platforms

**Fig. 2:**
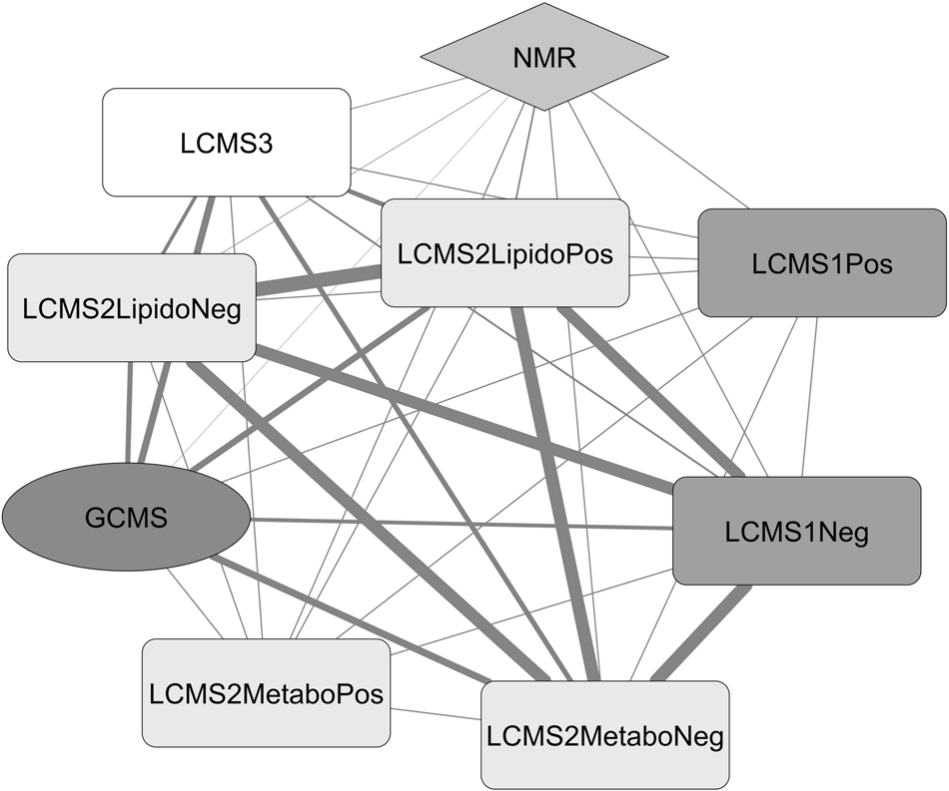
**Inter-platform correlation network**. The network was established using the RV scores between all the data matrices generated by the 5 analytical platforms. The thickness of the links reflects the concordance level in metabolite identification between platforms. The LC-MSn°1 was used in the positive (LCMS1Pos) or negative (LCMS1Neg) modes; the LC-MSn°2 was used in the metabolomics positive (LCMS2MetaboPos) or negative (LCMS2MetaboNeg) modes or in the lipidomics positive (LCMS2LipidoPos) or negative (LCMS2LipidoNeg) modes

In order to compare the results from all platforms, we developed a specific strategy to standardize the data. First, the LC-MS (Fig. 1A, B), GC-MS (Fig. 1C), and NMR (Fig. 1D) raw data were collected and standardized by the amounts of proteins. The analytical drift was then corrected using Workflow4Metabolomics, operating under the Galaxy environment, by a Van Der Kloet transformation^14^. As each data table contained intensities expressed as arbitrary units specific for each instrument, we used a log10 and then a Pareto standardization for data acquired by mass spectrometry and only Pareto for NMR data. The NMR results were somewhat distinct from those of the MS platforms, but the PCA analysis showed that NMR data were consistent with the (control, docetaxel) vs. (lovastatin, lovastatin + docetaxel) discrimination also observed by the MS platforms (Fig. 1d). Among the NMR signals, some were identified while others, identified by both GC-MS and LC-MS, remained unseen by NMR (Supplementary Table 1). That NMR- and MS-based metabolomics results were not entirely overlapping could result from the different physico-chemical properties of the compounds, or be linked to the fact that MS often detects minor species inaccessible to NMR. The data matrices that correlated most with each other were those from the LC-MSn°2 platform: RV_(LC-MSn°2LipidoPos_vs_LC-MSn°2LipidoNeg)_ = 0.978 and RV_(LC-MSn°2LipidoPos_vs_LC-MSn°2Neg)_ = 0.886, RV_(LC-MSn°2LipidoNeg_vs_LC-MSn°2Neg)_ = 0.870 (Fig. 2).

Metabolomics data generated by the same platform but in different ionization modes (positive or negative) showed partial correlation, such that the intra-platform RV was 0.257 on the LC-MSn°1 platform, and 0.342 on the LC-MSn°2 platform, even though the data were obtained from the same samples with a single extraction process. The data matrix size could explain the low correlation scores. Indeed, on the LC-MSn°1 platform, 1598 signals were extracted from the raw data in the positive mode, but only 613 signals in the negative mode. These low correlations scores likely reflected the fact that metabolomics extractions were more efficient for polar metabolites, which were separated by acidified phases in liquid chromatography, promoting a better ionization in the positive ion mode, including adducts, and thus, producing more detectable ions.

### Integration of metabolomics and lipidomics data

**Fig. 3:**
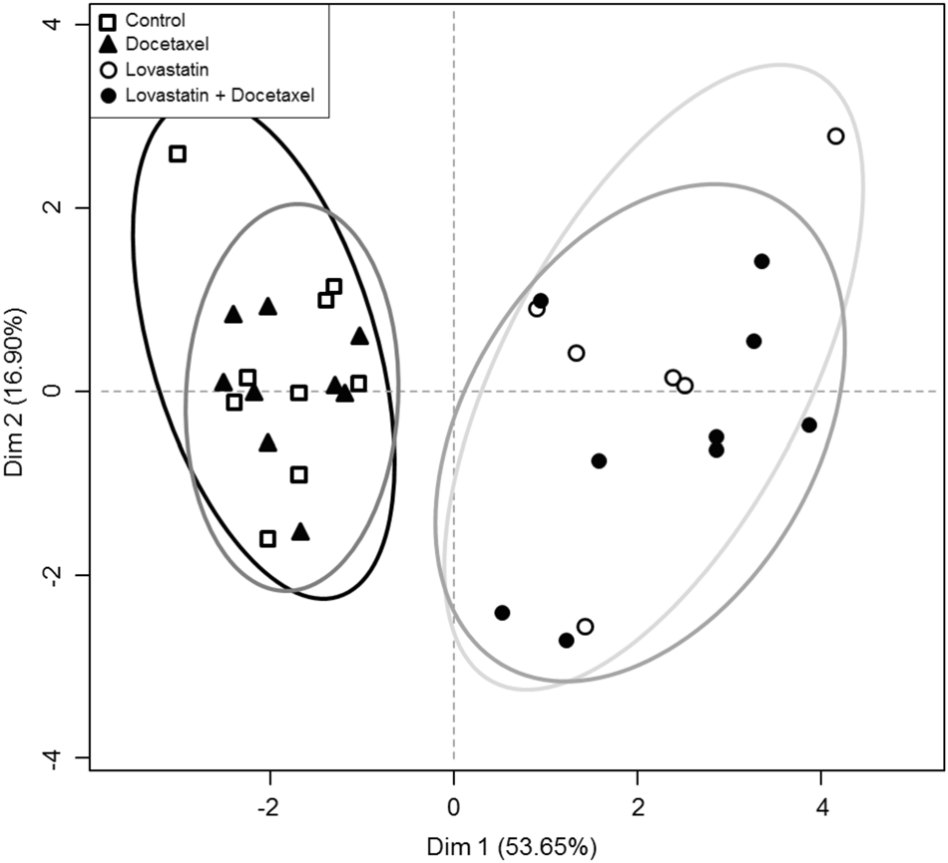
**Score plot of the “meta” PCA prepared from the calculated Dim1 coordinate from primary PCA analyses resulting from each data matrix**. Experimental conditions: control (open squares), docetaxel (plain triangles), lovastatin (open circles) and (lovastatin + docetaxel) (plain circles). Cells were treated for 36 h. The two dimensions of the analysis and their contribution to the variance are indicated

To address the consequences of the variations detected by the platforms, we performed PCAs for each platform, focusing on the coordinates from the first component of the PCAs, which explained most of the variance. We used these PCAs as information compression to reduce a matrix of data to a vector (defined by the coordinates of the sample in the first component) (Supplementary Figure 2). Using the first two components, the PCA explained 70% of the variance and, importantly, discriminated two large data sets (control, docetaxel) vs. (lovastatin, lovastatin + docetaxel) (Fig. 3). Hence, the global information obtained by each platform was convergent, and each platform would be able to determine whether a sample was treated at least by lovastatin. However, it was almost impossible to distinguish a control from a docetaxel-treated sample, or a lovastatin from a (lovastatin + docetaxel)-treated sample. However, our metabolomics approach also identified discrete and discriminant biomarkers.

### Metabolites and lipids identification

To determine the nature of the metabolites and lipids specifically altered by each treatment condition, several databases were queried, including the local database (NIST), online databases (LipidMaps, MassBank, HMDB) or in-house databases^15^. One hundred and eleven distinct compounds—some being detected by more than one platform, including amino acids, small organic acids, sugars, spermidine, phosphatidyl-choline or triglycerides—were sorted out across the nine data matrices generated by all platforms (Table 1). Depending on the metabolite considered, changes in its intracellular content varied specifically with the treatments. For instance, glutamine was increased in lovastatin or (lovastatin + docetaxel) extracts. Spermidine was decreased with (lovastatin + docetaxel). This dedicated biomarker identification allowed to better discriminate the different treatments than the global analysis of metabolomics profiles.

**Table 1:** Evolution of the 111 metabolites identified in the (lovastatin + docetaxel) vs. control condition after 36 h of treatment

Up				Down
2,3-Dihydroxypropyl palmitate	Cis-4-hydroxy-d-proline	L-Glutamine	Phosphoric acid	Choline
2-Hydroxy-3-Methylbutyric acid	Citric acid	L-Homoserine	Propionyl-L-carnitine	Fumarate
2-Oxovaleric acid	Creatine	L-Isoleucine	Pyridoxal	L-Glutathion
3-Hydroxytyramine	Creatinine	L-Leucine	Pyridoxine	L-Tryptophan
3-Methyl-2-oxobutanoic acid	Cyclohexene-3,5-dione	L-Lysine	Pyroglutamic acid	Pantothenic acid
4-Methyl-2-oxovaleric acid	Cystathionine	L-Methionine	Pyrophosphate	Spermidine
5-Aminovaleric acid	Dehydroabietic acid	L-Phenylalanine	Ribothymidine	Threonine
5-Deoxy-5-methylthioadenosine	D-Fructose	L-Proline	Sarcosine	SM 16:0
5-Methylcytidine	D-Glucose	L-Tyrosine	Stearoylglycerol	SM 18:0
Acetate	Dihydrouracil	Maleic acid	Succinate	SM 20:0
Adenosine	D-Ribose	Methylmalonic acid	Talose	SM 22:0
Adipic acid	Formamide	Monopalmitoylglycerol	Taurine	SM 24:0
Alpha-hydroxyisobutyric acid	Fructose di-phosphate	Myo-inositol	TG (52:2)	SM 18:1
Alpha-ketoglutaric acid	Glutamate	Myristic acid	TG (52:3)	SM 20:1
Aminoadipic acid	Glyceraldehyde	*N*-acetyl-asp-Glu	TG (54:2)	SM 22:1
Aminomalonic acid	Glycerol stearate	*N*-acetyl-d-glucosamine	Tryptophan	SM 24:1
Arginine	Glycerylphosphorylcholine	*N*-acetyl-l-aspartate	Tyramine	PC (28:0)
Asparagine	Hexadecanoic acid	*N*-acetyl-l-glutamate	Uric acid	PC (30:1)
Aspartate	Hippuric acid	Nicotinamide	Uridine	PC (32:2)
Azelaic acid	Hydroxybutyric acid	*O*-acetyl carnitine	Uridine-5′-monophosphate	PC (34:3)
Beta-Alanine	Hypotaurine	Ornithine	Valine	PC (34:5)
Betaine	Lactic acid	*O*-succinyl-l-homoserine		
Cer(d18:1/16:0)	L-Alanine	Phosphoglyceric acid		

HGT-1 cancer cells were treated for 36 h and collected for biochemical analyses. The identified metabolites (fold-change >2, *p*-value<0.05) are listed in alphabetical order, both in the “Up” and in the “Down” columns

**Fig. 4:**
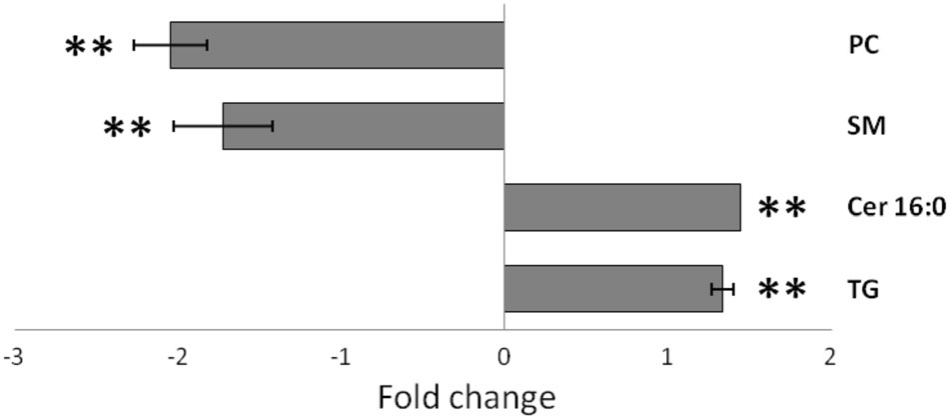
**Changes in lipid content**. Sphingomyelins (SM), ceramide (Cer 16:0), triglyceride (TG), and phosphatidylcholine (PC) fold-changes in cells treated with (lovastatin + docetaxel) vs. control. (**p-value<0.01). Fold changes (− or +) of the various lipid classes were represented as histogram bars. Cells were treated for 36 h

It was expected that levels of some lipids should be modified in response to statins. We thus analyzed intracellular lipidomes by two of the platforms (LC-MSn°2 and LC-MSn°3), using the Bligh & Dyer extraction procedure^16^. Four classes of metabolites were identified as significantly separating out the (lovastatin + docetaxel) condition from the control or docetaxel groups (Fig. 4): ceramide [Cer(d18:1/16:0)], phosphatidylcholines [PC(28:0), PC(30:1), PC(32:2), PC(34:3) and PC(34:5)], triglycerides [TG(52:2), TG(52:3) and TG(54:2)] and sphingomyelins [SM(16:0), SM(18:0), SM(20:0), SM(22:0), SM(24:0), SM(18:1), SM(20:1), SM(22:1), and SM(24:1)]. The amount of PC(30:1) and PC(32:2), detected in the positive mode, was reduced by (lovastatin + docetaxel). Conversely, [Cer(d18:1/16:0)] was increased in all cases. From the 19 triglycerides species detected, only 3 were modulated between treatment groups. No significant differences were observed between treatments for the sum of all detectable triglycerides, except a small decrease for docetaxel alone as compared to (lovastatin + docetaxel) (Supplementary Figure 3).

### Specifics of the analytical technologies

**Fig. 5:**
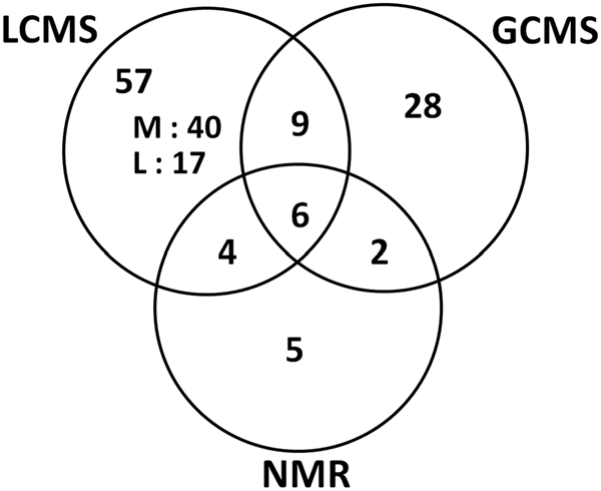
**Venn diagram of the specific and shared metabolites (M) and lipids (L)**. Metabolites detected by each technology and lipids detected by the LC-MS technology in the (lovastatin + docetaxel, 36 h) condition vs. control (fold change >1.5, *p*-value<0.05). The six metabolites common to LCMS2 and NMR were: glutamate, glutamine, myo-inositol, creatine, lactic acid, and fumarate

**Fig. 6:**
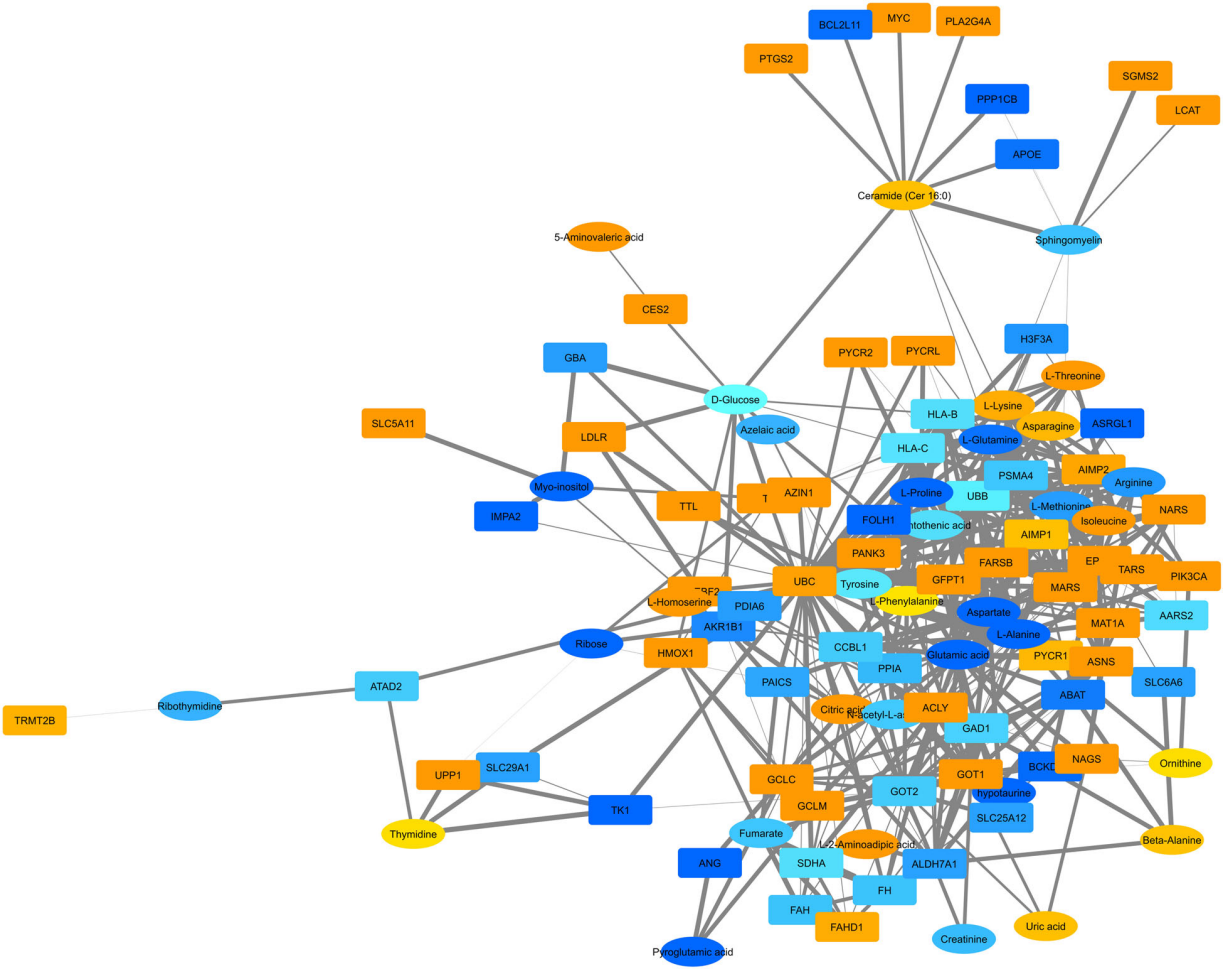
**Combined network analysis of metabolites and lipids**. Data from both the metabolomics and the lipidomics analyses were combined. Samples used were from (lovastatin + docetaxel)-treated cells for 36 h. The shapes and colors were as follows: ovals for metabolites and rectangles for lipids; from yellow to dark orange, more and more up-regulated, from light blue to dark blue, more and more down-regulated

Various compounds were identified by several platforms. Hence, in addition to the ability of the platforms to obtain a common descriptive envelope of the metabolome, we could also identify changes in the metabolomics content with the same accuracy, thereby conferring both convergence and robustness. In contrast, some compounds were detected by only one platform (e.g. acetate, choline, or formamide identified by NMR). Nevertheless, the unique ability of a given platform to identify specific metabolites added complementary information. To analyze the overlap in metabolites detected by all technologies, we established a Venn diagram (Fig. 5). Among all of the 111 identified biomarkers, six were common: glutamate, glutamine, myo-inositol, creatine, lactic acid, and fumarate (Table 2). Moreover, 12 metabolites were detected both by GC-MS and LC-MS, but not by NMR, including citric acid, pantothenic acid, glucose, l-proline, or l-methionine (Supplementary Table 1). Finally, we combined the metabolomics and lipidomics data and generated a network (Fig. 6). Although ceramides were modestly linked to other members of the network, a strong density of partner compounds was observed, stressing the close interplay between these lipid classes and the other biochemical categories of metabolites.

**Table 2:** Univariate quantitative analysis of metabolites commonly detected by all platforms

	GCMS		LCMS		NMR	
	Fold change	*t*-test	Fold change	*t*-test	Fold change	*t*-test
Creatine	2.05	0.0015	2.50	0.0476	2.17	0.0003
Fumarate	2.56	0.0143	2.34	0.0000	1.53	0.0114
Glutamate	1.72	0.0013	1.98	0.0000	1.53	0.0096
Glutamine	6.52	0.0009	7.12	0.0000	6.60	0.0129
Lactic acid	3.08	0.0038	3.96	0.0058	4.30	0.0275
Myo-inositol	2.56	0.0011	1.61	0.0220	1.68	0.0034

HGT-1 cancer cells were treated for 36 h and collected for biochemical analyses. The identified metabolites are listed in alphabetical order. The fold-changes and the *p*-values (Student *t*-test, *p* < 0.05) are shown

### Kinetic effects

**Fig. 7:**
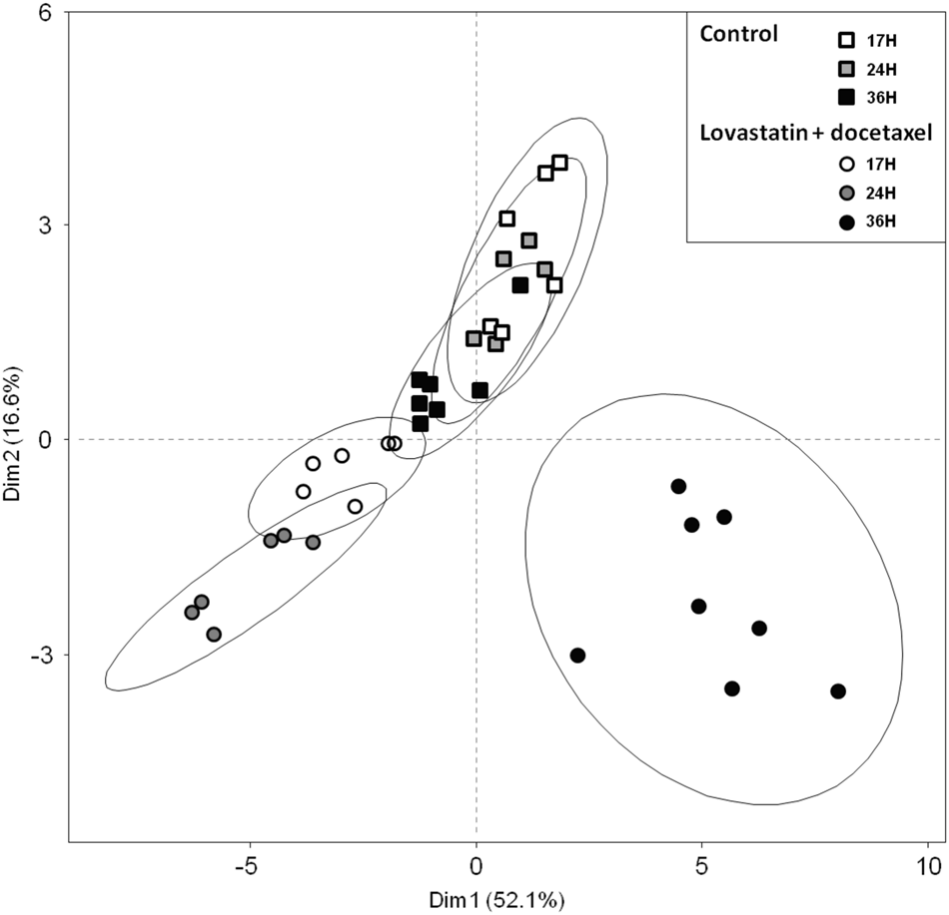
**PCA analysis of the kinetic effects of the (lovastatin** **+** **docetaxel) combination.** The cells were treated with both drugs for the indicated times (H) and cell extracts were prepared for GC-MS analysis. Seventeen-hours control (open squares), 24 h control (gray squares), 36 h control (black squares), 17 h treament (empty circles), 24 h treatment (gray circles), and 36 h treatment (black circles). Three biological replicates of each treatment condition were performed

We used quantitative GC-MS data for these experiments. Each set of data was normalized with respect to its own control at the same time of treatment. We analyzed 31 metabolites from cells treated by (lovastatin + docetaxel) for all time-points. The (lovastatin + docetaxel) treatments were best separated from their respective 17 h and 24 h controls according to the first dimension of the PCA (Fig. 7), like for cells treated by lovastatin alone at the same time points (data not shown). The first dimension of the PCA, which explained the largest fraction of the variance, showed a clear separation of the (lovastatin + docetaxel) condition at 36 h, from either the control or the drug combination at earlier time-points. Strikingly, nearly all metabolites were increased for the 36 h time-point, suggesting increased synthesis over time (Supplementary Table 2). By contrast, fumarate and pantothenic acid levels were decreased at all time-points.

### Overlap between transcriptome changes and metabolites/lipids altered levels

Metabolites with a fold change >1.5 and a *p*-value <0.05 (lovastatin + docetaxel condition vs. control) and genes with a fold change >2 (lovastatin + docetaxel condition vs. control) were integrated into a global network using the Stitch software^17^ for interaction analysis of metabolites and proteins, and the Cytoscape environment for visualization^18^. This highlighted links between “cholesterol homeostasis” and “cellular carbohydrate biosynthetic processes” through intermediates from the “response to transition metal particles” and “female gonad development”. “Cellular amino acids metabolic processes” appeared unlinked to these pathways (Supplementary Figure 4).

We next used the ClueGO^19^ application that extracts the non-redundant biological information for large clusters of genes, and PaintOmics^20^, which permits overlay transcriptomics and metabolomics data onto KEGG pathways. We identified several significantly affected pathways (Supplementary Information and Supplementary Figures 5–10). While most metabolites were increased, fumarate was decreased. Interestingly, expression of several genes from these pathways was either increased or decreased by the treatment (Supplementary Figures 5, 7–10), whereas one pathway showed no modifications in gene expression, while displaying metabolite changes (Supplementary Figure 6).

**Fig. 8:**
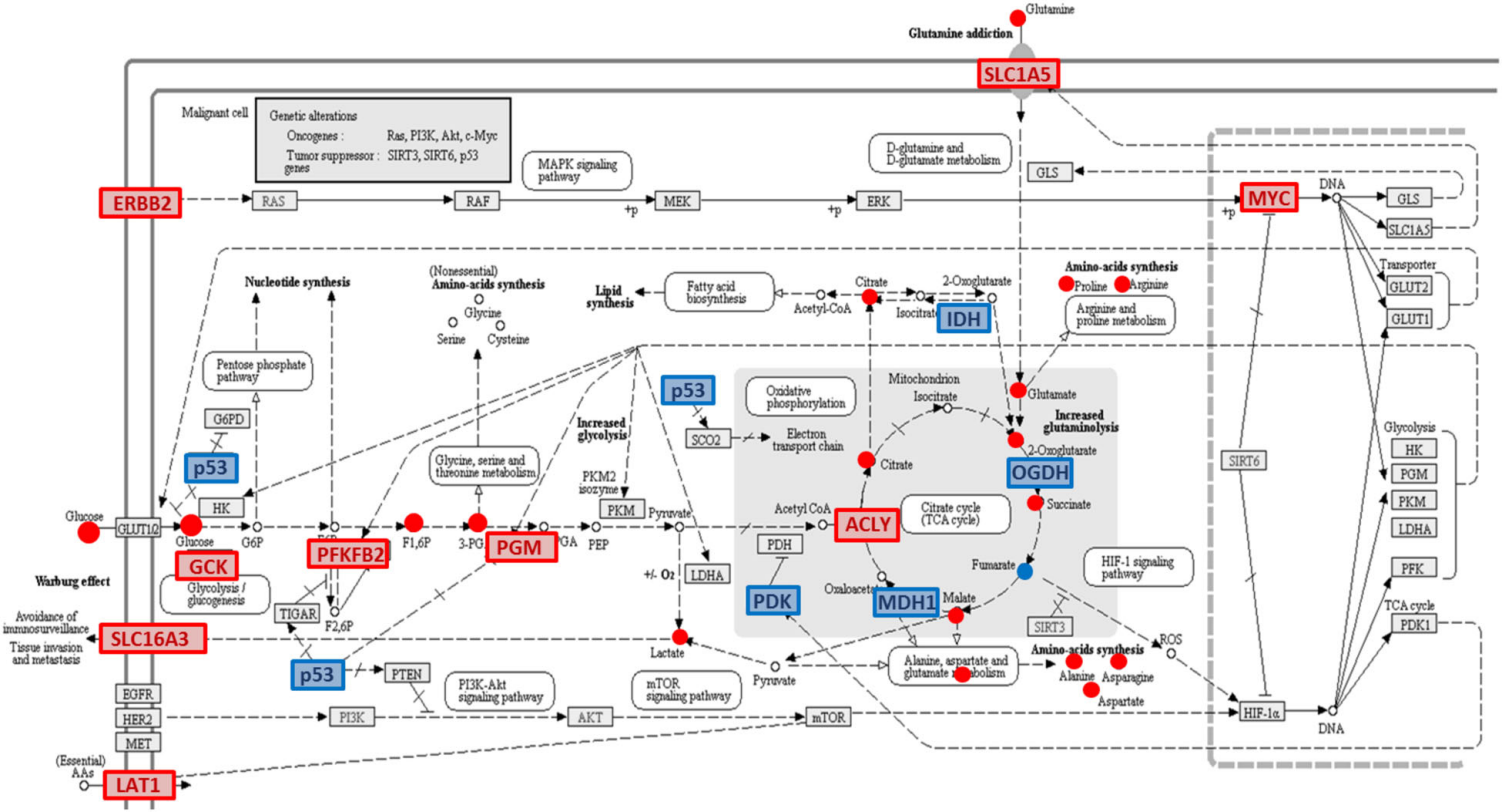
**Central carbon metabolism in cancer**. Metabolite and gene expression changes in response to (lovastatin + docetaxel) treatment for 36 h. The metabolomics and lipidomics signaling pathways were overlaid with the alterations in gene expression following (lovastatin + docetaxel) treatment compared to control cells after 36 h. The color code was as follows: blue boxes and blue circles referred to down-regulated genes and down-regulated metabolites, respectively. Red boxes and red circles referred to up-regulated genes and up-regulated metabolites, respectively

Finally, integrating our data into the general pathway of “Central carbon metabolism in cancer” (http://www.genome.jp/kegg-bin/show_pathway?hsa05230) highlighted several genes that were up-regulated by the (lovastatin + docetaxel) treatment, including the *ACLY*, *ERBB2*, *GCK*, *MYC*, *PKFB2*, *SLC1A5*, *SLC7A5*, and the *SLC16A3* genes, together with several modified metabolites identified by one or more platforms, such as amino acids, citrate, lactate, fumarate, 2-oxoglutarate or succinate. Importantly, several other genes were down-regulated, like *P53*, *IDH*, *OGDH*, *MDH1*, and *PDK*. From these observations, it can be anticipated that glucose uptake, amino acid synthesis and lactate production be increased, together with a drop in the P53-dependent sensitization to apoptosis and a stimulation of cell division through activation of the ERBB2-dependent MAP kinase pathway (Fig. 8).

## Discussion

The identification of biomarkers of response to anticancer treatments is particularly important to predict patient outcome. Model cancer cell systems are one such tool to decipher the effects of treatments onto cellular metabolic activity. In a previous study, we identified the (lovastatin + docetaxel) combination as a novel means to trigger human HGT-1 gastric cancer cells apoptosis quite efficiently^1^. Here, we set out to establish a robust description of metabolomics/lipidomics alterations in response to the drugs, as an initial step towards further biomarker characterization. As such, identified metabolites (Table 2) will be further explored as coherent biomarkers of drug response.

To address the possibility that the metabolome and the lipidome of gastric cancer cells in vitro could show specific alterations in response to lovastatin and/or docetaxel, we selected independent MS and NMR platforms. There was a good concordance between the results from these platforms that identified both a comparable trend of variations, and several of the same metabolites, as especially shown by the clear distinction between treatments containing or not lovastatin. Importantly, a similar discrimination between the control and the (lovastatin + docetaxel) conditions was also observed here in two other cancer cell lines from the stomach and the colon (Supplementary Figure 1). Almost all metabolite levels decreased during the first 24 h, but rose afterwards, at 36 h of treatment, possibly indicating a synthesis rebound phase (Supplementary Table 2). However, both fumarate and pantothenic acid levels were decreased at all time points.

The metabolism of lovastatin has been amply described^21–23^. Docetaxel is extensively metabolized by hepatic cytochromes P-450 (CYP), particularly CYP3A and CYP2C^24^ in human liver. The identified metabolites and lipids whose levels were modified in response to the drugs were distinct from the metabolic products of lovastatin and docetaxel. Several metabolites from the tricarboxylic acid (TCA) cycle were raised, indicating that its activity was higher than in control cancer cells, hence closer to that of normal cells. We might suggest that this “normal-like” metabolic condition may be incompatible with the mutations or epigenetic changes that drove cancer cells to switch from oxidative phosphorylation to aerobic glycolysis, participating in apoptotic cell death in response to the drugs.

Several lipid classes were altered by the treatments, including phosphatidylcholines and sphingomyelins (decreased), ceramides, and triglycerides (increased). Reportedly, lovastatin decreased phosphatidylcholine concentration by inhibiting cytidylyltransferase activity In human endothelial cells^25^. Ceramides, which have a well-defined pro-apoptotic role, are highly produced during apoptosis, mostly through the action of sphingomyelinase, and induce sphingomyelinase, which converts sphingomyelin back into ceramides^26,27^, leading to amplifying apoptotic signals in cells treated by anticancer agents^28,29^. The rises observed here were likely attributable, at least in part, to the apoptosis-inducing ability of lovastatin, or to the statin-dependent increase in ceramide synthase^30^.

Docetaxel binds tubulin, thereby preventing microtubule repolymerization, provoking mitotic arrest and cell death^31^. It decreased expression of several genes in breast cancer cells, including the *BCLX* and *BCL2* anti-apoptotic genes^32^. In addition, paclitaxel, another taxane, down-regulated several lipid synthesis genes in ovarian cancer, including the *APOE*, *HMGCS1*, and *LDLR* genes, indicating that docetaxel could also contribute to alterations of lipid levels^33^. In paclitaxel-treated HeLa cells, a drop in phosphatidylcholine was observed^13^, which also occurred here in docetaxel-treated HGT-1 cells.

Lovastatin increased metabolites from the TCA cycle in cancer cells (from the ovary), but reduced the metabolites associated with glycolysis. Nearly all, among metabolites involved in the TCA cycle, together with amino-acids, were up-regulated similarly by lovastatin in this study^34^ and by (lovastatin + docetaxel) in our study, including citrate, lactate, malate, succinate, tryptophan, and valine.

We showed previously that treatment with lovastatin and/or docetaxel engaged cells from several cancer types into apoptosis, in addition to gastric cancer cells, including cervix, lung, or liver cancer cells^1^. Because most of the metabolomics and lipidomics changes observed in response to (lovastatin + docetaxel) could not be distinguished from those resulting from lovastatin alone, we surmise that any lovastatin-responsive cancer cell type should show comparable metabolomics and lipidomics profiles.

To determine the links between both metabolomics/lipidomics and gene expression data types, we used PaintOmics^20^. Several of the biochemical pathways showing variations in activity were indeed associated with selected changes in mRNA levels (see Supplementary Information and Supplementary Figures 5, 7–10). Although most modifications in biochemical pathways were associated with alterations in gene expression, other altered pathways were apparently unlinked to gene expression changes, possibly because their variation in expression remained undetectable, or because the cell response to (lovastatin + docetaxel) did not require adjustment of mRNA levels of genes from the considered pathways, e.g. for the “amino acids biosynthesis” pathway (Supplementary Figure 6).

Importantly, glucose and glutamine levels, two major sources of energy for cancer cells^3,35^, were increased, so was citrate—a precursor of fatty acids—and lactate—a promoter of invasive potential. Hence, the (lovastatin + docetaxel) treatment of HGT-1 cells did not oppose the normal regulation of energy supply characteristic of cancer cells. Rather, it amplified production or capture of metabolites important for cancer cell metabolism. Because these metabolites’ levels dropped at the early time-points, their further rise at 36 h could be interpreted as a rebound phase, which was, however, insufficient to allow cell survival, but possibly pointing to the ability of these cells to try developing survival strategies.

In view of the multiplicity of alterations occurring both at the transcriptome and metabolome/lipidome levels, and not knowing if these were a driver or a consequence of apoptosis engagement, or a mixture of both, it will be difficult to obtain a fair appreciation of which of these altered pathways would be best targeting for tumor growth inhibition in vivo. Provided the observed association between metabolite and gene variation levels were linked, at least in part, it could be envisioned to modulate expression of some of the genes deregulated under our pro-apoptotic conditions to directly address their roles in cell death. Although it may seem counterintuitive that apoptosis resulting from the (lovastatin + docetaxel) treatment was associated with a drop in *P53* gene expression, this might be unlinked to HGT-1 sensitivity to apoptosis since these cells carry an inactivating *P53* mutation. Furthermore, we observed that *P53*-proficient HCT116 colon carcinoma cells were also highly sensitive to the (lovastatin + docetaxel) combination, indicating that the apoptosis-inducing capacity of these drugs may not require either active P53 proteins or, possibly, induction of some of their targets (e.g. pro-apoptotic Bcl-2 family members). We are currently addressing these important questions.

## Conclusion

Our results, based on a thorough approach that mobilized several independent technological platforms, demonstrated that the treatment of gastric cancer cells by lovastatin +/− docetaxel triggered profound metabolomics and lipidomics alterations at the beginning of overt apoptosis. Although the sugar dependency and consumption by cancer cells has been amply reported, our data stressed the fact that some metabolomics modifications that accounted for altered glucose metabolism could also occur in response to lipid-restricting drugs like statins. We identified several cases of overlap between metabolomics/lipidomics and transcript levels, indicating that some of the biochemical changes could have been contributed by modifications in gene expression. It also appeared that the pro-apoptotic effects of the (lovastatin + docetaxel) combination were able to surpass the rise in energy supply that would otherwise be sufficient to fulfill the metabolic needs of cancer cells. We speculate that targeting energy sources may not necessarily be sufficient to kill cancer cells in the environment of a living organism. Rather, inhibiting anti-apoptotic proteins at the same time may be a more efficient option. It may also be suggested that some of the metabolites/lipids—and the genes that participated in producing them—that were modified, could provide novel biomarkers, or possibly pertinent targets, involved in the response to apoptosis-promoting treatments in a more general sense.

## Material & methods

### Cell culture and treatments

HGT-1 human gastric cancer cells were grown in 90 mm plates at 37 °C under a humidified atmosphere of 5% CO_2_ in DMEM (Dulbecco’s modified Eagle’s medium) (Lonza, Saint Beauzire, France), containing 4.5 g L^−1^ glucose and supplemented with 5% fetal bovine serum without antibiotics (Gibco-Invitrogen, Cergy-Pontoise, France)^12,36^. AGS and HCT116 human gastric cancer and colon cancer cells, respectively, were grown under the same conditions in DMEM containing 4.5 g L^−1^ glucose and supplemented with 5% fetal bovine serum without antibiotics. The medium was changed at day +2 and day +4. At day +4, the culture medium was replaced by DMEM with L-glutamine, without phenol red, for the time of treatment, as it would otherwise saturate the signal, particularly in liquid chromatography-mass spectrometry (LC-MS). Cells were treated at 80% (roughly 10 million cells) cell confluence, with lovastatin (12.5 μM, final concentration) and / or docetaxel (5 nM) for up to 36 h. DMSO (Dimethyl sulfoxide), the vehicle of lovastatin, was added to all cell plates at the same concentration (less than 0.5% V/V) with no apparent toxicity observed in DMSO only-treated cells used as controls. At the morphological level, docetaxel-treated cells showed no alterations. By contrast, lovastatin or (docetaxel + lovastatin)-treated cells did show signs of initiating apoptosis (cell blebs) after 36 h whereas no such signs were observed after 24 h. Adherent cells were rinsed twice with 10 mL cold phosphate buffered saline and 5 mL of ice-cold methanol were added per dish to stop any metabolic process and lyse the cells in situ. Cells were then centrifuged and the cell pellets were rinsed with 1 mL of ice-cold methanol. Pellets were subjected to sonication on ice (2 × 15 s at 70 Hz) and distributed into aliquots. For protein assays, 0.1 mL was collected. All the platforms received 1.5 mL cell pellet fractions. Three independent biological replicates were prepared several weeks apart, and sent under the same conditions to all platforms.

### Determination of HMG-CoA reductase activity

Cell pellets (4 million cells) were dissolved in 200 µl buffer containing 50 mM NaH_2_PO_4_, 10 mM EDTA, 0.1 mM DTT (pH 7.4). Leupeptin (10 µg/mL) and PMSF (1 µL/mL of 1 M PMSF in DMSO) were added just before use. Cells were disrupted by 20 passages in a glass/glass dounce followed by 20 s sonication. After a 15 min centrifugation at 1500×*g*, the supernatants were removed and stored at −80 °C until use. Protein concentration was determined according to Bradford^37^.

HMG-CoA Red was measured essentially as described^38^ with some modifications. Incubation mixtures (200 µL) contained buffer (100 mM KH_2_PO_4_, 50 mM KCl, 1 mM EDTA, and 5 mM DTT, pH 7.4), 1.7 nmol of [^14^C]HMG-CoA and 8.3 nmol of HMG-CoA (final concentration of 50 µM). The enzyme reactions were started by the addition of 200 µg of proteins and 2.5 mM NADPH. After 60 min incubation at 37 °C, reactions were terminated by adding 30 µL of 72% trichloracetic acid. After 30 min to allow conversion of mevalonate to mevalonolactone, samples were centrifuged 3 min at 16 000×*g* and 40 µL of supernatant were used for HPLC analysis. In each sample, the HMG-CoA Red activity was determined in duplicate.

Samples (30 µL) were injected and separated on a reverse phase Nucleosil C18 column (5 µm particle size, 250 × 4.6 mm equipped with a 5 mm guard column of the same phase) with the following linear gradient of potassium phosphate 50 mM pH6.8 (solvent A) and methanol (solvent B): 5% B for 2 min, 5–32% B up to 16 min, 32–5% for 10 min, at a flow rate of 0.8 mL/min. The HPLC chromatograph was interfaced with a Flo-one Beta radiometric detector (Packard, Meriden, USA). Peak areas were calculated from the percentage of metabolite area to the total product area (metabolite + residual substrate). Data were expressed as pmol/min/mg of protein.

### Metabolomics and lipidomics analytical methods (see Supplementary information)

Five instrumental platforms contributed to the analyses: NMRn°1 (1H-NMR, Bruker), LC-MSn°1 (UHPLC-LTQ-Orbitrap, Thermo), LC-MSn°2 (UHPLC-Exactive, Thermo), LC-MSn°3 (UPLC-HRMSe, Synapt Q-TOF G2, Waters), GC-MSn°1 (GC-MS, Agilent). From extraction to analysis, each analytical platform processed the samples using its in-house procedure (Supplementary Table 3). Altogether, the analyses, i.e. metabolomics, lipidomics, in both positive or negative ionization modes (for MS), resulted in 9 data matrices from 5 platforms. Each platform generated its own dataset in a tabular format where variables were the peak area of a m/z index for the mass spectrometry analysis or a bucket for NMR. These datasets included the 3 replicates for each treatment (control, lovastatin, docetaxel and co-treatment lovastatin + docetaxel), the analytical blank samples, and quality control samples (QC). QC samples were generated from an in-house protocol in varying numbers depending on each platform (none for NMRn°1, 10 for LC-MSn°1, LC-MSn°2 and GC-MSn°1, 9 for LC-MSn°3). They were prepared by making a mixture of all samples (controls and tests). They were used to correct the analytic drift, to filter the data and to evaluate statistics validity (reproducible profiles). They were introduced as follows: 5 at run start, then every 5–10 samples, depending on the separation sequence, and 3 at the end of the run (for GC). The relative standard deviation (RSD, %) was calculated for QC variables peak areas to evaluate their analytical quality and robustness. Finally, lipid markers having a RSD value below 30% in QC samples were kept for subsequent multivariate analysis. For the kinetics study, 34 selected biomarkers were quantified by GC-MS following the procedure described for the GC-MSn°1 platform. Sphingomyelins and ceramides were quantified in samples by a validated assay on the LC-MSn°3 platform, as described previously^39^. Additional platform protocols are presented in Supplementary Information.

### Data analysis

Nine data matrices were generated by XCMS included in the workflow4metabolomics. We have collected all raw data from each platform, and we then have tuned each extraction parameter as finely as possible: one metabolomics data matrix in the positive electronic impact mode for GC-MSn°1, two metabolomic data matrices obtained in the positive and the negative ESI mode for LC-MSn°1, two metabolomic data matrices and two lipidomic data matrices generated in the positive and the negative ESI mode for LC-MSn°2, one lipidomic data matrix obtained in the positive ESI mode for LC-MSn°3 and one metabolomic data matrix for NMRn°1 platform. For each of the data matrices generated, a batch correction was applied to minimize the signal drift considering the inherent QCs.

Then, to compare these data matrices, a normalization step was introduced, i.e. combining all the generated data to be analyzed in the same order of magnitude. Mass spectrometry data were first normalized by a log_10_ transformation, then all datasets (including MS and NMR) were Pareto transformed^13^.

The relationship between the data tables from the different platforms were analyzed using the RV coefficient^40,41^. This RV coefficient can be interpreted as a multivariate equivalent of the coefficient of determination, which is a measure of the quality of the prediction of a linear regression (R²) ranging from [0–1]. When considering two matrices X and Y, a coefficient RV equaling to 1 means that the relative position of the samples in X is similar to that in Y. In other words, the information contained in the two tables is identical. For each data matrix generated, a principal component analysis (PCA) was also performed with FactoMineR and the FactoExtra package working under the R computing environment^42^. The coordinates of the first dimension (Dim 1) of each PCA describing a data matrix were combined into a single file. We felt that multivariate tests would provide a fair description of the phenomena. Nevertheless, all univariate comparisons were also performed, which showed the same results, i.e. the equivalence of the control and docetaxel condition, the equivalence of the lovastatin and lovastatin + docetaxel condition, and the clear separation of the control and lovastatin, the control and lovastatin + docetaxel, the docetaxel and the lovastatin, the docetaxel and the lovastatin + docetaxel conditions. Univariate determinations are reported in Supplementary Tables 1 and 2. It could be observed that all platform determinations were quite homogeneous, i.e. obtained similar trends of variations for these metabolites. In addition, glutamine was the most strongly increased metabolite in response to the combination of lovastatin and docetaxel. However, some even stronger increases occurred for a few metabolites detected by a given platform, LCMS vs. GCMS, e.g. citric acid (10.22-fold according to LCMS, but 1.75-fold according to GCMS) or glutathione (4.55-fold according to LCMS and 2.1-fold according to GCMS). All statistical experiments were also performed with FactoMineR or Workflow4Metabolomics 3.0 (workflow4metabolomics.org).

## Electronic supplementary material

Supplementary information

PCA analysis of two additional cancer cell lines

Diagram of the “meta” PCA protocol

Comparison of total triglycerides levels

Cluego analysis of the biological pathways affected by the drugs

Alanine, aspartate and glutamine metabolism (KEGG) pathway

Biosynthesis of amino acids (KEGG) pathway

Pantothenate and CoA biosynthesis (KEGG) pathway

Butanoate metabolism (KEGG) pathway

Sulfur metabolism (KEGG) pathway

Arginine metabolism (KEGG) pathway
