# Supplementary material for: Consequences of blunting the mevalonate pathway in cancer identified by a pluri-omics approach
Source: Cell Death Dis. 2018 Jul 3;9(7):745. doi: 10.1038/s41419-018-0761-0 (PMC6030166; doi:10.1038/s41419-018-0761-0)
Supplement: Supplementary file 1 — Supplementary information [file 41419_2018_761_MOESM1_ESM.docx]

**Supplementary information**

**Platform protocols**

*GC-MSn°1 (INSERM Brest): GC/MS metabolomic approach*

To 700 μL of cell pellets frozen in methanol, 10 μL of 2 mM adonitol were added as internal standard and vortexed for ten seconds for extraction. Derivatization was carried out by adding 30 μL of MeOx methoxyamine (20 mM in pyridine), incubation for 90 min at 30 °C, and by adding 80 μL of Sylon [BSTFA-TMCS (99: 1)] incubated at 37 °C for 30 min. After allowing the samples to stand for 2 hours at room temperature, 1 μL was injected in GC-MS (6570-5973N, Agilent) in the EI mode (70eV) equipped with an HP-5MS column (30m * 0.25mm ID * 0.25μ J & W Scientific).

Data were extracted, normalized and annotated using the workflow http://workflow4metabolomics.org/ running under Galaxy (parameters: xcmsSet centwave; ppm 100; peakwidth 5,25; mzdiff 1; snthreshold 6; integrate 1; prefilter 3,100; 5000; group density; minfrac 0; bw 20; mzwid 0.25; max 100; retcor obiwarp; profStep 1). Statistical analyzes were conducted under this environment, and also under the FactoMinor package running under R environment. The identification was confirmed by comparison with the mass spectra from the NIST bank 08 or with the mass spectra of the local mass spectra database.

*LC-MSn°1 (UPMC/CNRS Roscoff): UHPLC/LTQ-Orbitrap metabolomic approach*

Extraction was carried out on 1 mL MeOH per cell pellet, addition of 200 μL H_2_O and on 400 μL supernatant addition of 1600 μL MeOH so as to obtain a ratio [MeOH /H_2_O (80:20, v:v)]. Samples were stirred for 1 hour at 4°C and then centrifuged at 14000 rpm for 10 min before transfer into LC/MS vials. Ten μL were injected into LC-MS on a C18 acclaim 100 * 2.1 mm * 1.7 μ (Dionex) column mounted on a Dionex U3000 RSLC. A mobile phase gradient was applied, starting at 5% B for 3 min, then 100% B over 10 min maintained for 5 min, then returning to the initial conditions by stabilizing for 5 min (A: H_2_O 0.1% acetic acid, B: Acetonitrile 0.1% acetic acid, flow rate: 400 μL / min). The samples were analyzed in positive and negative modes on an LTQ-Orbitrap (Thermo) (50-1000 *m/z*; R = 30000 @ *m/z* 400).

Data were pre-processed, normalized, and annotated using the workflow http://workflow4metabolomics.org/ running under Galaxy environment (parameters: xcmsSet centwave; ppm 5; peakwidth 5,40; mzdiff -0,001; snthreshold 6; integrate 1; prefilter 3,100, group density, minfrac 0.5, bw 10, mzwid 0.25, max 50, retcor obiwarp, profStep 1). Statistical analyses were also carried out under this environment as well as under SIMCA-P + 12 software (Umetrics).

*LC-MSn°2(LABERCA, Nantes): UHPLC/Exactive metabolomic and lipidomic approach*

This platform carried out two types of analyses: a metabolomic analysis and a lipidomic analysis. The supernatant metabolome was analyzed following extraction of 400µl by 1400 μL MeOH. After stirring for 15 s, the samples were centrifuged at 15800 g for 20 min at 10 ° C. 370 μL of the methanol phase were collected, dried and suspended in 100 μL H_2_O. Lipidomic analysis was carried out on 60 μl of supernatant extracted with 220 μL of MeOH (vortex 10 s), 750 μL MTBE (vortex 10 s) and 188 μL H_2_O. After stirring, the samples were centrifuged at 14000 g for 2 min at 10 °C. Three hundred μL of the MTBE phase were removed and evaporated to dryness and then taken up in 70 μl of a mixture [ACN/IPA/H_2_O (65/30/5)]. For intracellular analysis, about 1.5 mL of cell pellet frozen in MeOH was extracted with 3040 μL of chloroform, vortexed for 10 s, supplemented with 960 μL H_2_O, vortexed for 20 s. The samples were centrifuged at 8000 g for 10 min at 10 °C. The [MeOH/H_2_O] phase was dedicated to metabolomic analysis and the chloroform phase was reserved for lipidomic analysis. The samples were characterized on an HPLC-HRMS instrument (Exactive-Thermo) in dual polarity mode (ESI + / ESI-), scan range: 65-1000 *m/z*; R = 25000 @ *m/z* 400. The R software with the XCMS package was used for data processing. Data filtration was performed according to RT, EIC and CV of the QC. Statistical analyzes were run under SIMCA-P (Umetrics).

*LC-MSn°3 (CRNH, Nantes): UHPLC/Synapt-G2 lipidomic approach*

One hundred µL of cell pellets frozen in methanol were suspended in 100 μL H_2_O. The extraction was carried out according to the Bligh & Dyer method by adding 800 μL of a mixture [MeOH/CHCl_3_ (2/1, v/v)] to the 200 μL of the suspended pellets or supernatant. After stirring, the samples were centrifuged for 10 min at 10,000 rpm and then dried under nitrogen at room temperature. The pellet was taken up in 150 μL of an IPA/ACN/H_2_O mixture (2/1/1, v/v/v). The extracted metabolites were injected (10 μL) and analyzed in UHPLC-HRMS^E^ positive and negative modes on an Acquity CSH C_18_ column: 1.7 μm, 2.1 × 100 mm mounted on an Acquity H-Class UPLC system (Waters) coupled to a Q-TOF Synapt G2 HDMS (Waters, R = 20000 @ *m/z* 556). Data analysis was conducted under MarkerLynx (Waters) and the univariate statistical analyses under GraphPad Prism 4. The lipids were identified by their elemental composition (*m/z* to ± 10 ppm), the query of the LIPIDMAPS online database and their fragmentation schemes.

*NMRn°1 (CEISAM, Nantes): NMR metabolomic approach*

On the samples of MeOH-fixed pellets, a biphasic extraction [MeOH, CHCl_3_, H_2_O (5: 5: 3.6)] was carried out. After centrifugation at 5000 rpm for 15 min at 4 °C, the aqueous phase was removed and dried under a stream of nitrogen. The pellet was suspended in 595 μL of deuterated borate buffer (63 mM, pH 10 in D_2_O) and 5 μL of 2 mM trimethylsilylpropanoic acid (TSP). After stirring, the sample was transferred to an NMR tube for analysis. The pH was readjusted directly into the tube in order to have a better alignment of the spectra at all the metabolites with NaOD and DCL (max volume added 4 μL, therefore less than 0.7% of the initial volume). The apparatus used was a Bruker Avance HD NMR Spectrometer operating at a ^1^H frequency of 700.28 MHz and equipped with an inverse ^1^H/^13^C/^15^N/^2^H cryogenically cooled probe. Data were acquired at 298 K with a standard 1D pulse sequence including continuous presaturation of the water signal during the recovery delay. The free induction decays (FIDs) were recorded over 12.0 ppm with 8 scans and 4 dummy scans, a recovery delay of 5 s and an acquisition time of 2.6 s. The FIDs were apodized with a 0.3 Hz exponential decay and zero-filled to 128 k data points before Fourier transform. The spectra were then manually phased and baseline corrected. The statistical analyzes were carried out under the SIMCA-P + 12 (Umetrics) software. The biomarkers were identified from the literature ([1](#_ENREF_1)).

**Overlap between biochemical pathways and gene expression alterations**

The alteration in activity of the “alanine, aspartate and glutamate” metabolism pathway was paralleled by reduced expression of the *GOT1* gene (glutamic-oxaloacetic transaminase 1), an enzyme involved in amino acid metabolism and the urea and tricarboxylic acid cycles, and of the *GFPT1* gene encoding the glutamine-fructose-6-phosphate transaminase 1 that catalyzes the formation of glucosamine 6-phosphate and controls the flux of glucose into the hexosamine pathway. The *GOT1* gene was also involved in the arginine synthesis pathway. In addition, the *FOLH1B* gene (folate hydrolase 1B) and the *ABAT* gene (4-aminobutyrate aminotransferase), responsible for the catabolism of gamma-aminobutyric acid, were both up-regulated. These two genes were also expected to contribute to the activity of the “alanine, aspartate and glutamate” pathway. Over-expression of the *ABAT* and *BDH2* genes (3-hydroxybutyrate dehydrogenase 2) gene was associated with deregulation of the butanoate metabolism pathway. By contrast, the *HMGCS1* gene (3-hydroxy-3-methylglutaryl-CoA synthase 1), involved in the early step of cholesterol synthesis, was down-regulated. The *PANK* gene (pantothenate kinase 3), which participates in the synthesis of coenzyme A, was down-regulated and associated with variation in activity of the “pantothenate and coenzyme A synthesis” pathway. The *SUOX* gene encoding the sulfite oxidase, which catalyzes the oxidation of sulfite to sulfate, the final reaction in the oxidative degradation of cysteine and methionine was up-regulated and associated with the “sulfur metabolism” pathway. Finally, the *NAGS* gene encoding the N-acetylglutamate synthase involved in forming N-acetylglutamate from glutamate and Acetyl CoA, was up-regulated and associated with alterations of the “arginine biosynthesis” pathway.

**Supplementary Table 1**: ***Univariate determination of variant metabolites***

|  | **GCMS** | | **LCMS** | | **NMR** | |
| --- | --- | --- | --- | --- | --- | --- |
|  | Fold change | T.test | Fold change | T.test | Fold change | T.test |
| Lactic acid | 3.08 | 0.0038 | 3.96 | 0.0058 | 4.30 | 0.0275 |
| Creatine | 2.05 | 0.0015 | 2.50 | 0.0476 | 2.18 | 0.0003 |
| Glutamine | 6.52 | 0.0009 | 7.12 | 0.0000 | 6.60 | 0.0129 |
| Glutamate | 1.72 | 0.0013 | 1.98 | 0.0000 | 1.53 | 0.0096 |
| Myo-inositol | 2.56 | 0.0011 | 1.61 | 0.0219 | 1.68 | 0.0034 |
| Fumarate* | 2.56 | 0.0143 | 2.34 | 0.0000 | 1.53 | 0.0114 |
| L-Methionine | 2.60 | 0.0023 | 2.02 | 0.0000 | - | - |
| L-Proline | 2.24 | 0.0013 | 2.64 | 0.0000 | - | - |
| Citric acid | 1.75 | 0.0114 | 10.22 | 0.0241 | - | - |
| Pantothenic acid* | 3.67 | 0.0274 | 2.53 | 0.0000 | - | - |
| Glucose | 2.03 | 0.0013 | 3.97 | 0.0051 | - | - |
| Homo-serine | 1.86 | 0.0031 | 2.48 | 0.0000 | - | - |
| Isoleucine | 2.19 | 0.0138 | 1.66 | 0.0047 | - | - |
| Tryptophan | 2.82 | 0.0000 | 2.19 | 0.0000 | - | - |
| Phenylalanine | 2.15 | 0.0029 | 2.08 | 0.0000 | - | - |
| Glutathion | 2.10 | 0.0164 | 4.55 | 0.0000 | - | - |

The variation levels (Student T.test) of the metabolites were determined in extracts from HGT-1 cells treated for 36h by (lovastatin + docetaxel). *All metabolites were induced, except fumarate and panthothenic acid, which were decreased. Significance levels figured as 0.0000 stand for p-values < 10^-4^.

**Supplementary Table 2**: ***Kinetics of variation of the 31 metabolites quantified by GC-MS***

|  | 17 h | 24 h | 36 h |
| --- | --- | --- | --- |
| Adenosine | 🡮 | 🡮 | 🡭 |
| Arginine | **-** | **-** | 🡭 |
| Asparagine | 🡮 | 🡮 | 🡭 |
| Aspartic acid | **-** | **-** | 🡭 |
| Azelaic acid | 🡮 | 🡮 | 🡭 |
| Beta-Alanine | 🡮 | 🡮 | 🡭 |
| Citric Acid | 🡮 | 🡮 | 🡭 |
| Creatinine | 🡮 | 🡮 | 🡭 |
| D-Glucose | 🡮 | 🡮 | 🡭 |
| Fumarate | 🡮 | 🡮 | 🡮 |
| Glutamic acid | **-** | **-** | 🡭 |
| Hypotaurine | **-** | **-** | 🡭 |
| L2-Aminoadipic acid | 🡮 | 🡮 | 🡭 |
| L-Alanine | 🡮 | 🡮 | 🡭 |
| L-Glutamine | **-** | **-** | 🡭 |
| L-Homoserine | 🡮 | 🡮 | 🡭 |
| L-Isoleucine | 🡮 | 🡮 | 🡭 |
| L-Lysine | 🡮 | 🡮 | 🡭 |
| L-Methionine | 🡮 | 🡮 | 🡭 |
| L-phenylalanine | 🡮 | 🡮 | 🡭 |
| L-Proline | 🡮 | 🡮 | 🡭 |
| L-Tyrosine | 🡮 | 🡮 | 🡭 |
| Maleic Acid | 🡮 | 🡮 | 🡭 |
| Myo-Inositol | 🡮 | 🡮 | 🡭 |
| N-Acetyl-L-aspartate | 🡮 | 🡮 | 🡭 |
| Ornithine | 🡮 | 🡮 | 🡭 |
| Pantothenic acid | 🡮 | 🡮 | 🡮 |
| Pyroglutamic Acid | 🡮 | 🡭 | 🡭 |
| Ribothymidine | 🡮 | 🡮 | 🡭 |
| Tyramine | 🡮 | 🡮 | 🡭 |
| Uric Acid | 🡮 | 🡮 | 🡭 |

HGT1 cancer cells were treated for the 17, 24 and 36 hours, and collected for biochemical analyses. The identified metabolites are listed in alphabetical order. 🡮 decreased level, 🡭: increased level, as compared to control condition. For this study, the 31 selected biomarkers were quantified by GC-MS following the procedure described for the GC-MSn°1 platform. Sphingomyelins and ceramides were quantified in samples by a validated liquid chromatography-tandem mass spectrometry (LC-MS/MS) assay described previously ([2](#_ENREF_2)). (-): no change.

**Supplementary table 3: *Specifics of the analytical platforms***

| **Plateform** | **Technology** | **Metabolomics** | **Lipidomics** | **Data processing** | **Identification** |
| --- | --- | --- | --- | --- | --- |
| GCMSn°1 | GC-MS (Simple Quadripole, Agilent) | OK | NO | workflow4metabolomics.org | NIST14, In-house database |
| LCMSn°1 | LC-MS (LTQ Orbitrap, Thermo) | ESI positive and Negative mode | NO | workflow4metabolomics.org | Massbank, GMD, In-house database |
| LCMSn°2 | LC-MS (Exactive, Thermo) | ESI positive and Negative mode | ESI Positive and Negative mode | XCMS/CAMERA | In-house database |
| LCMSn°3 | LC-MS (Q-TOF Synapt G2, Waters) | NO | ESI Positive and Negative mode | MarkerLynx | LipidMaps, In-house database |
| NMRn°1 | NMR (Avanced, Bruker) | OK | NO | TopSpin | Litterature |

**Legends to supplementary Figures**

**Supplementary figure 1**: ***PCA analysis of two additional cancer cell lines***. AGS human gastric cancer cells and HCT116 human colon cancer cells were treated for 36h by lovastatin and/or docetaxel. A PCA analysis was performed with samples analyzed by GC-MS, which showed a clear separation of the metabolomic responses under the (lovastatin + docetaxel) condition *vs.* the control condition. This was quite comparable with the results obtained for HGT-1 cells.

Score plot from PCA of the quantified amount of each biomarker for control condition (C) and the lovastatin + docetaxel (LD) treatment for each cell line (blue: AGS, red: HCT1161, green: HGT-1) Ellipses (in violet) describe 95% in the generated model.

**Supplementary figure 2: *Diagram of the “meta” PCA protocol***. PCAs were generated for each platform focusing on the coordinates from the first component of the PCAs, which explained most of the variance associated to our datasets. We reduced these PCAs data to a vector defined by the coordinates of the sample in the first component. We generated a new dataset to compare conditions (control, docetaxel, lovastatin, lovastatin + docetaxel).

**Supplementary figure 3: *Comparison of total triglycerides levels***. Triglycerides levels were determined by LC-MS. Almost no distinction was observed between the treatment groups (Kruskal-Wallis test).

**Supplementary Figure 4**: ***Cluego analysis of the biological pathways affected by the drugs*.** The analysis was performed with sample data from the metabolomics changes triggered by (lovastatin + docetaxel) after 36 hours, in comparison with untreated cells. Colors were set up by the application. Multicolored compounds belonged to several pathways. The sizes of the nodes reflected the enrichment significance of the terms.

**Supplementary figure 5**: ***Alanine, aspartate and glutamine metabolism (KEGG) pathway***. The pathway has been supplemented with up- (in red) and down- (in blue) regulated metabolites and gene expression.

**Supplementary figure 6**: ***Biosynthesis of amino acids (KEGG) pathway***. The pathway has been supplemented with up-regulated metabolites (in red).

**Supplementary figure 7**: ***Pantothenate and CoA biosynthesis (KEGG) pathway***. The pathway has been supplemented with up- (in red) and down- (in blue) regulated metabolites and gene expression.

**Supplementary figure 8**: ***Butanoate metabolism (KEGG) pathway***. The pathway has been supplemented with up- (in red) and down- (in blue) regulated metabolites and gene expression.

**Supplementary figure 9**: ***Sulfur metabolism (KEGG) pathway***. The pathway has been supplemented with up- (in red) and down- (in blue) regulated metabolites and gene expression.

**Supplementary figure 10**: ***Arginine metabolism (KEGG) pathway***. The pathway has been supplemented with up- (in red) and down- (in blue) regulated metabolites and gene expression.

**References**

1. Robert O, Sabatier J, Desoubzdanne D, Lalande J, Balayssac S, Gilard V, et al. pH optimization for a reliable quantification of brain tumor cell and tissue extracts with (1)H NMR: focus on choline-containing compounds and taurine. Analytical and bioanalytical chemistry. 2011 Jan;399(2):987-99. PubMed PMID: 21069302.

2. Croyal M, Kaabia Z, Leon L, Ramin-Mangata S, Baty T, Fall F, et al. Fenofibrate decreases plasma ceramide in type 2 diabetes patients: A novel marker of CVD? Diabetes & metabolism. 2017 May 09. PubMed PMID: 28499696.
