# Supplementary figures and images for: Consequences of blunting the mevalonate pathway in cancer identified by a pluri-omics approach
Source: Cell Death Dis. 2018 Jul 3;9(7):745. doi: 10.1038/s41419-018-0761-0 (PMC6030166; doi:10.1038/s41419-018-0761-0)

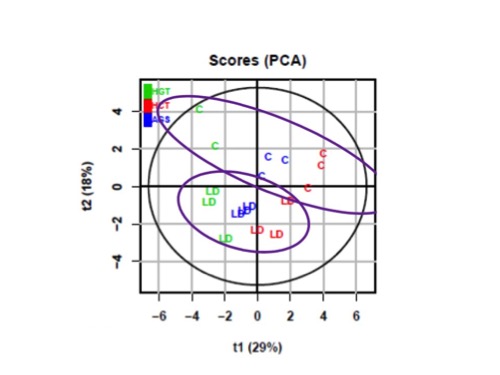

Supplement: Supplementary file 2 — PCA analysis of two additional cancer cell lines [file 41419_2018_761_MOESM2_ESM.jpg]

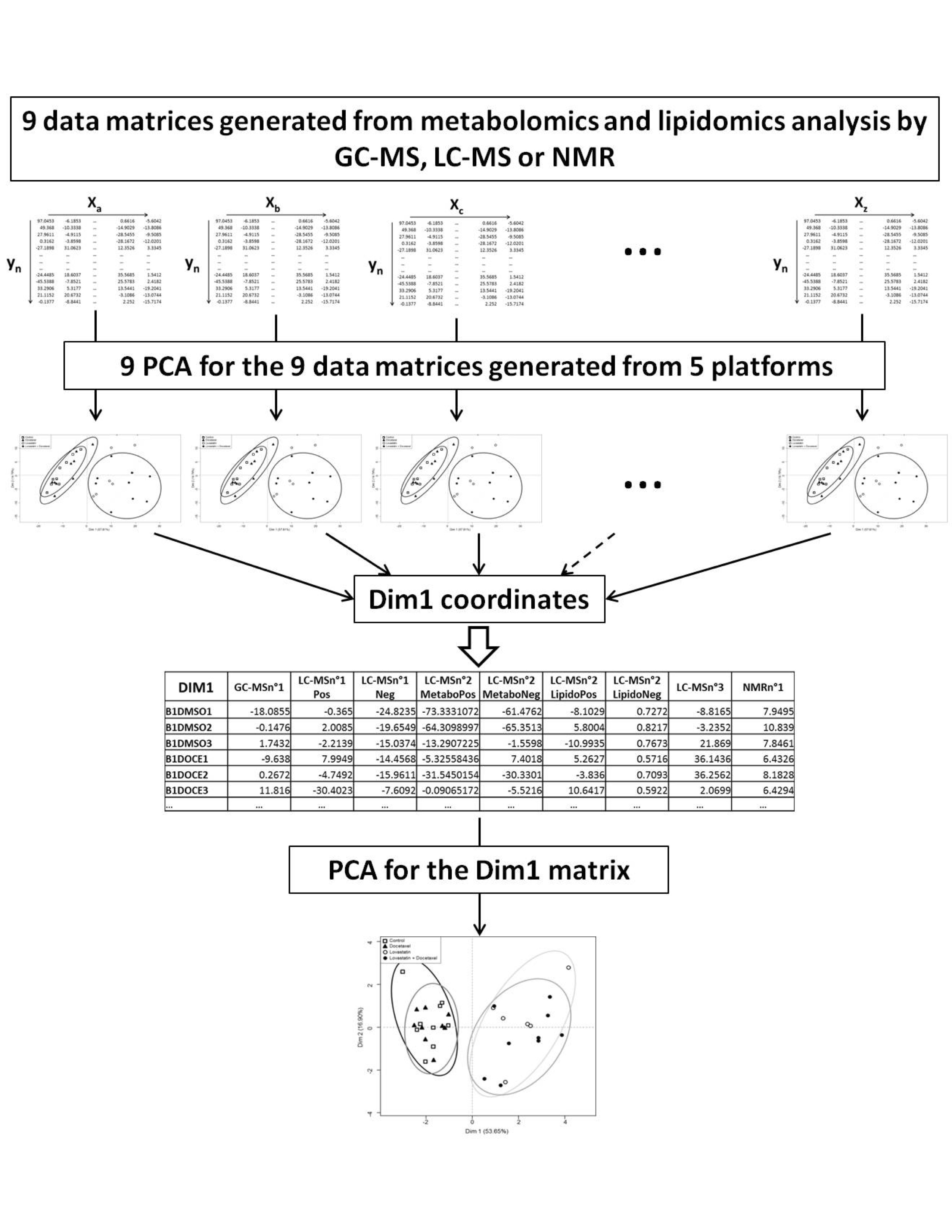

Supplement: Supplementary file 3 — Diagram of the “meta” PCA protocol [file 41419_2018_761_MOESM3_ESM.jpg]

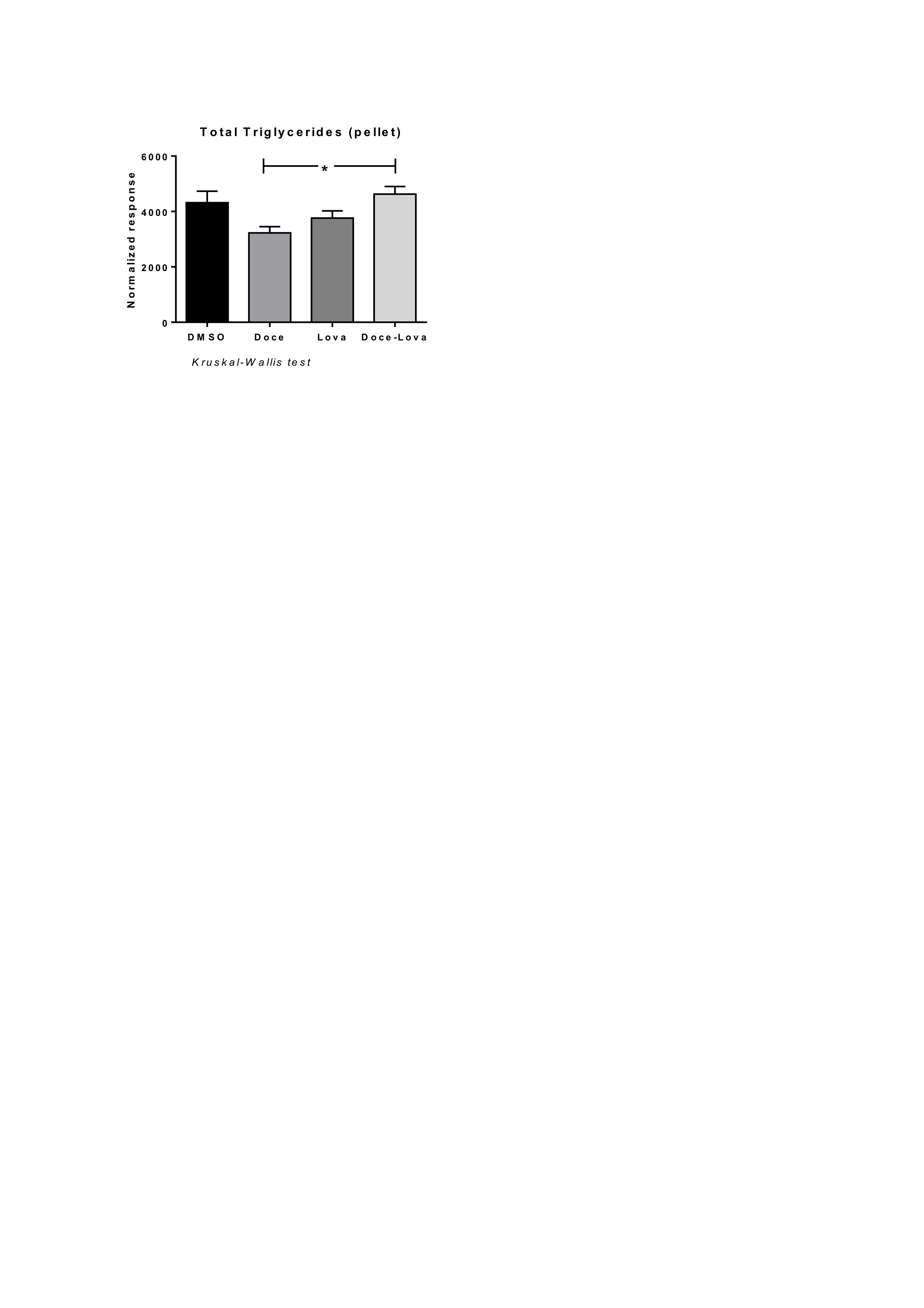

Supplement: Supplementary file 4 — Comparison of total triglycerides levels [file 41419_2018_761_MOESM4_ESM.jpg]

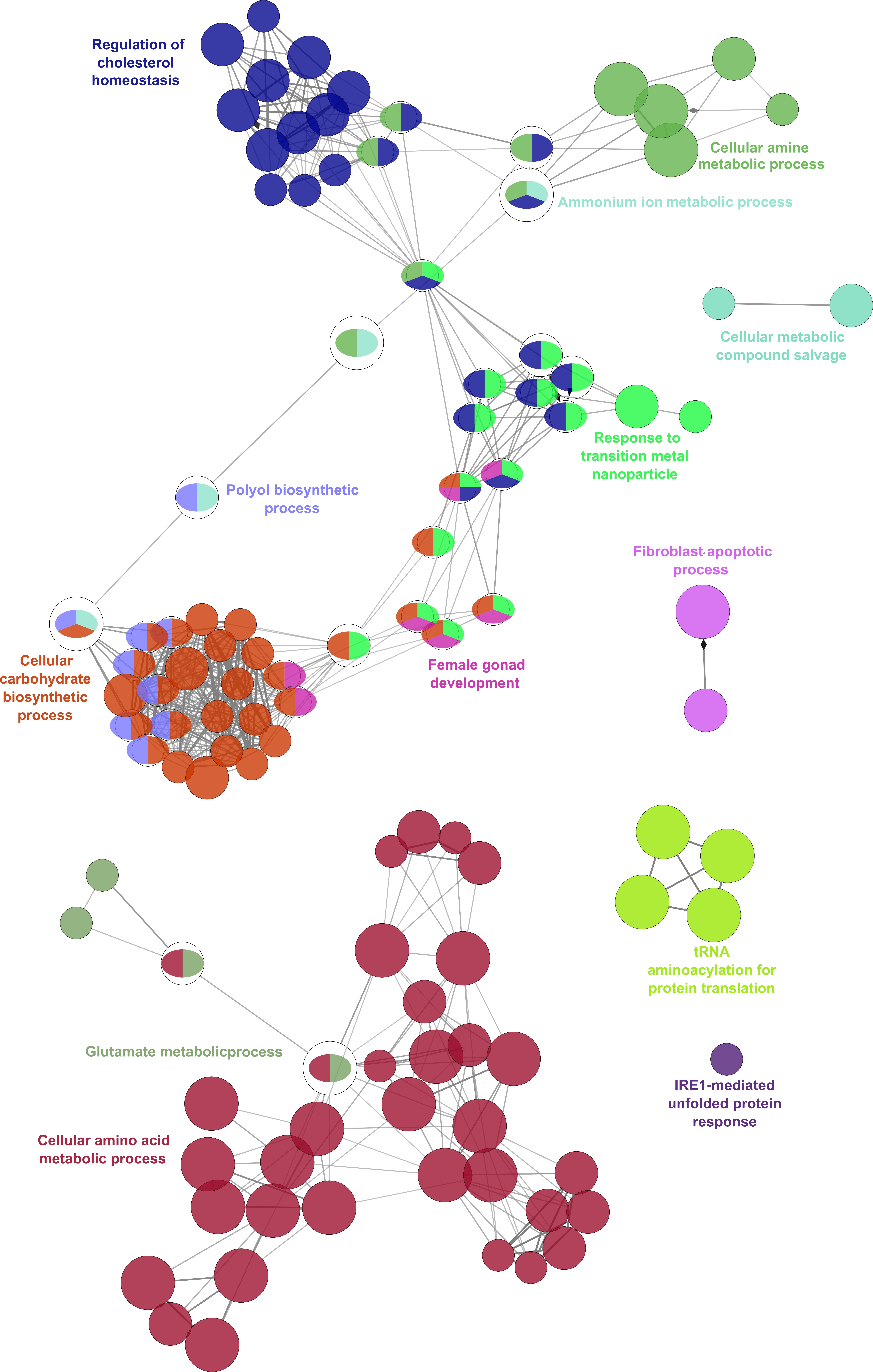

Supplement: Supplementary file 5 — Cluego analysis of the biological pathways affected by the drugs [file 41419_2018_761_MOESM5_ESM.jpg]

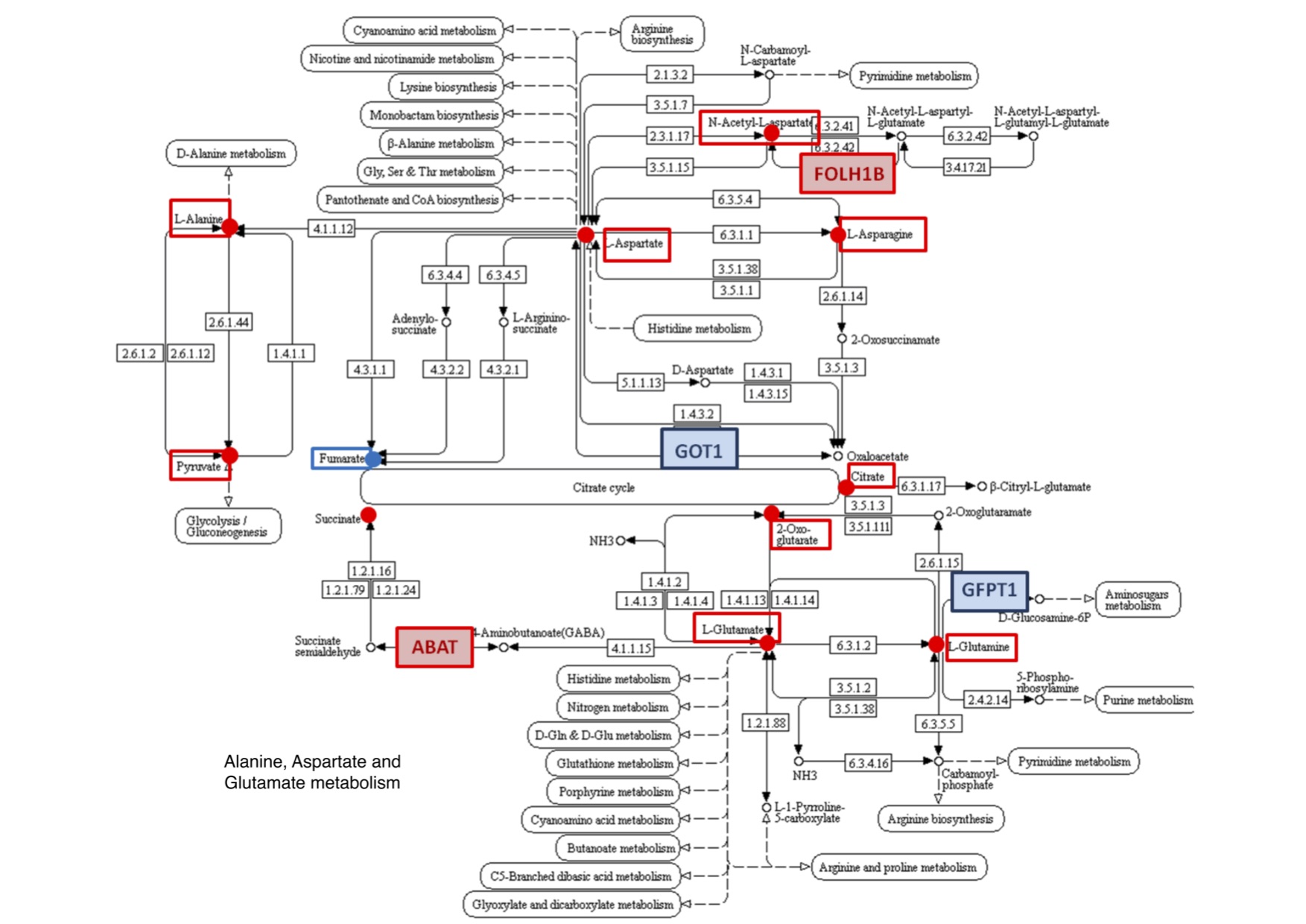

Supplement: Supplementary file 6 — Alanine, aspartate and glutamine metabolism (KEGG) pathway [file 41419_2018_761_MOESM6_ESM.jpg]

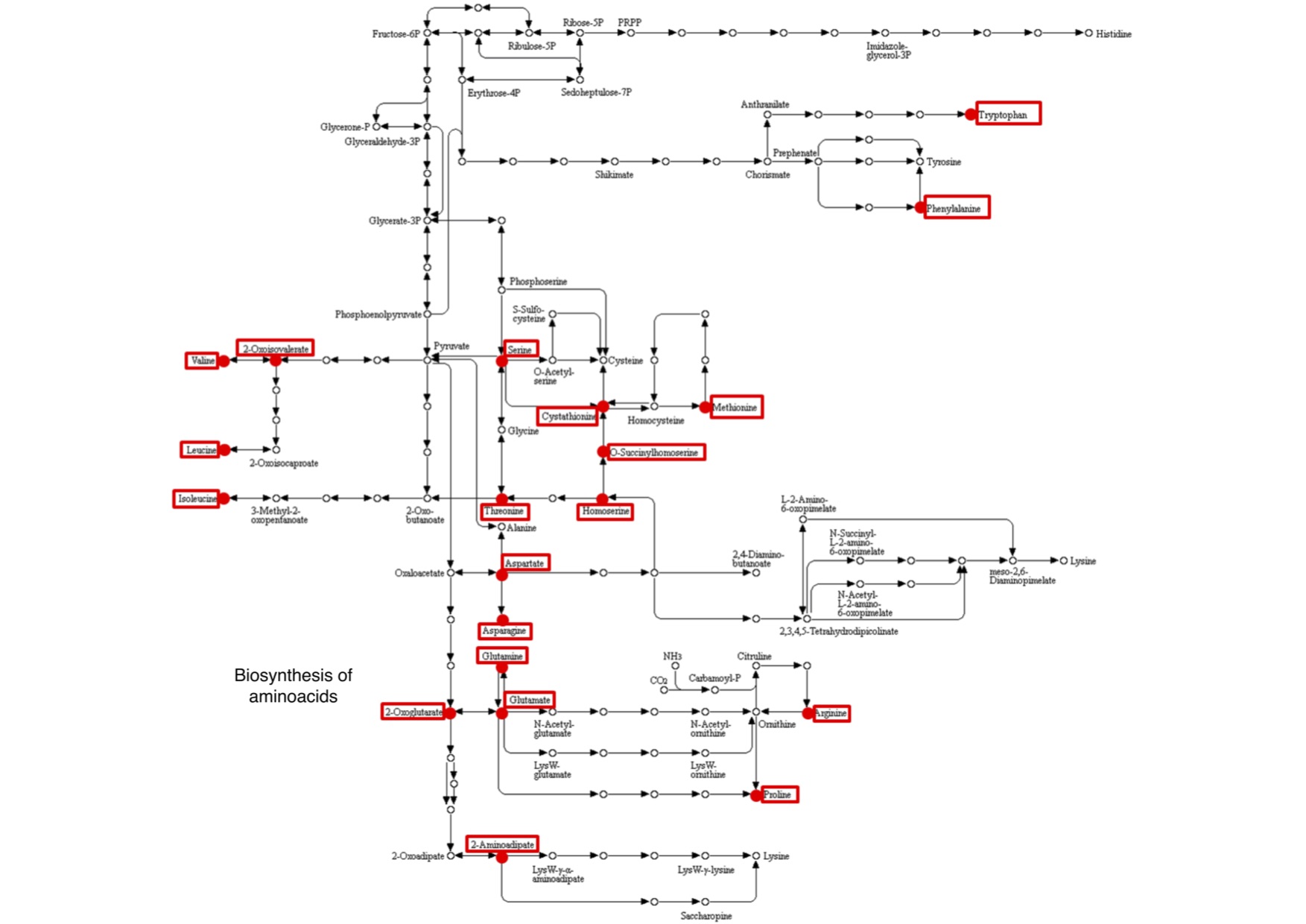

Supplement: Supplementary file 7 — Biosynthesis of amino acids (KEGG) pathway [file 41419_2018_761_MOESM7_ESM.jpg]

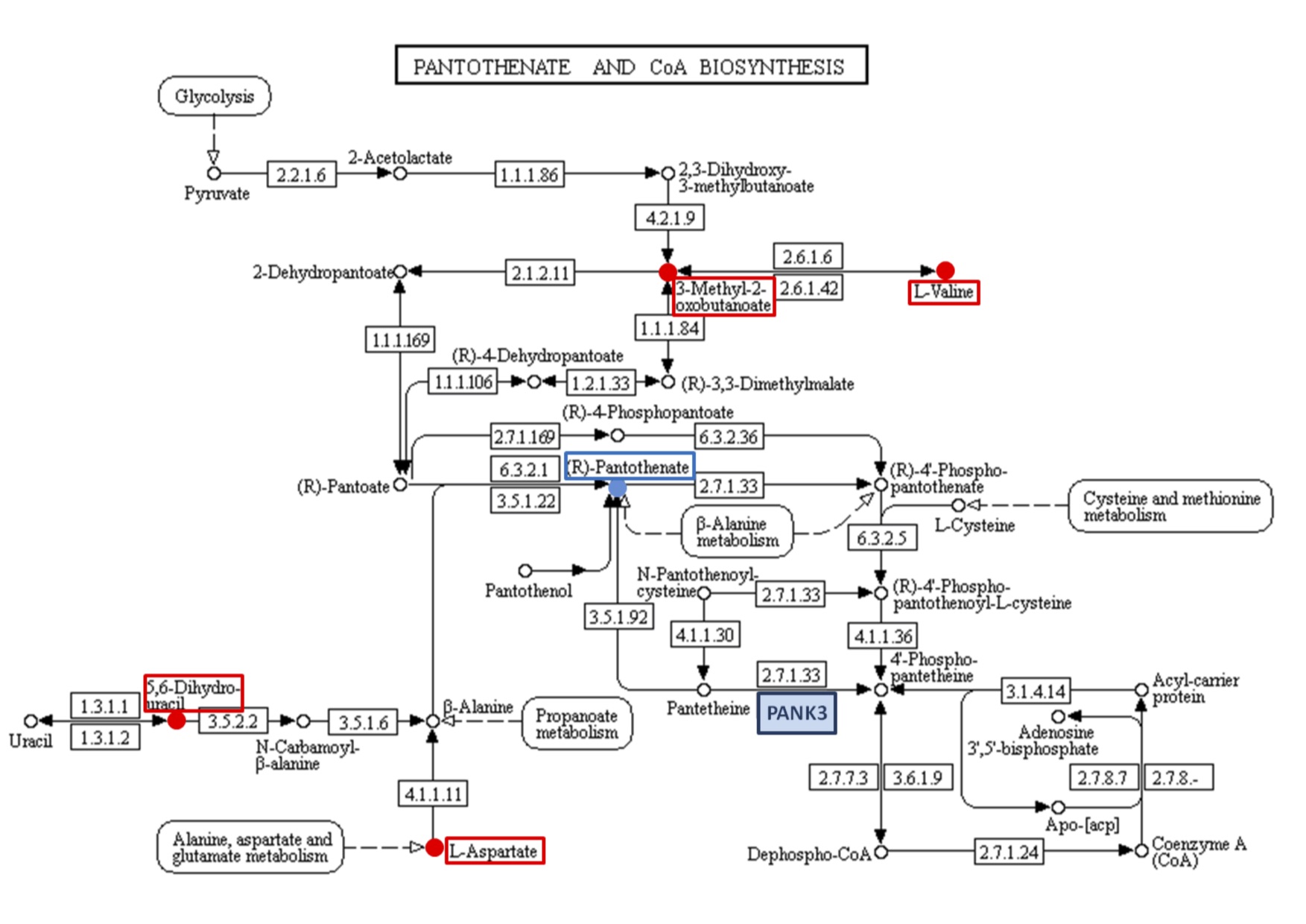

Supplement: Supplementary file 8 — Pantothenate and CoA biosynthesis (KEGG) pathway [file 41419_2018_761_MOESM8_ESM.jpg]

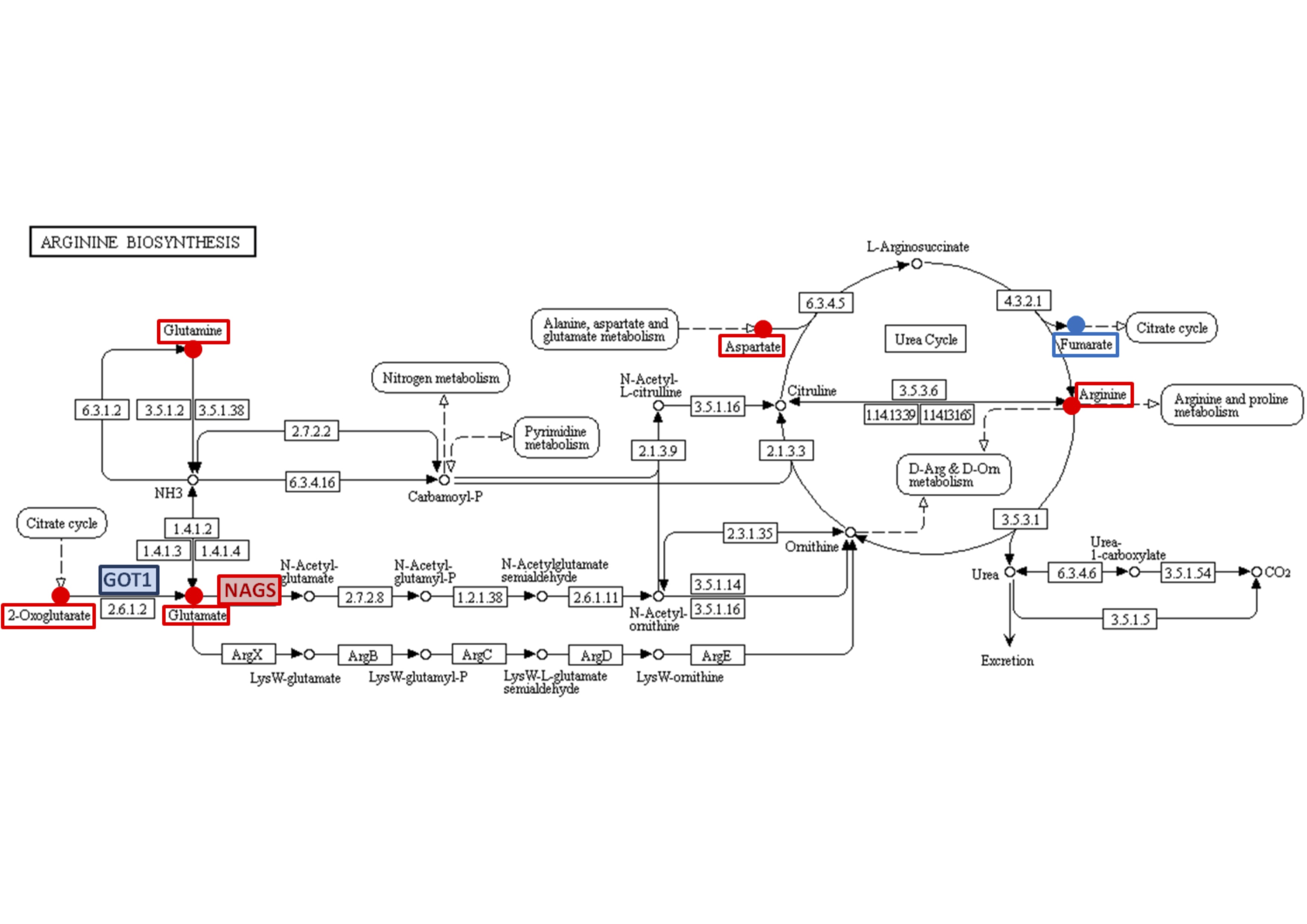

Supplement: Supplementary file 11 — Arginine metabolism (KEGG) pathway [file 41419_2018_761_MOESM11_ESM.jpg]
